# Toxic/Bioactive Peptide Synthesis Genes Rearranged by Insertion Sequence Elements Among the Bloom-Forming Cyanobacteria *Planktothrix*

**DOI:** 10.3389/fmicb.2022.901762

**Published:** 2022-07-28

**Authors:** Elisabeth Entfellner, Ruibao Li, Yiming Jiang, Jinlong Ru, Jochen Blom, Li Deng, Rainer Kurmayer

**Affiliations:** ^1^Research Department for Limnology, University of Innsbruck, Mondsee, Austria; ^2^Institute of Virology, Helmholtz Zentrum München, Munich, Germany; ^3^Department of Ecology and Institute of Hydrobiology, Jinan University, Guangzhou, China; ^4^Bioinformatics and Systems Biology, Justus-Liebig-University, Giessen, Germany

**Keywords:** harmful algal blooms, secondary metabolites, insertion sequence elements, microevolution, chromosomal rearrangements, genome size variation, horizontal gene transfer, cyanotoxins

## Abstract

It has been generally hypothesized that mobile elements can induce genomic rearrangements and influence the distribution and functionality of toxic/bioactive peptide synthesis pathways in microbes. In this study, we performed in depth genomic analysis by completing the genomes of 13 phylogenetically diverse strains of the bloom-forming freshwater cyanobacteria *Planktothrix* spp. to investigate the role of insertion sequence (IS) elements in seven pathways. Chromosome size varied from 4.7–4.8 Mbp (phylogenetic Lineage 1 of *P. agardhii*/*P. rubescens* thriving in shallow waterbodies) to 5.4–5.6 Mbp (Lineage 2 of *P. agardhii*/*P. rubescens* thriving in deeper physically stratified lakes and reservoirs) and 6.3–6.6 Mbp (Lineage 3, *P. pseudagardhii*/*P. tepida* including planktic and benthic ecotypes). Although the variation in chromosome size was positively related to the proportion of IS elements (1.1–3.7% on chromosome), quantitatively, IS elements and other paralogs only had a minor share in chromosome size variation. Thus, the major part of genomic variation must have resulted from gene loss processes (ancestor of Lineages 1 and 2) and horizontal gene transfer (HGT). Six of seven peptide synthesis gene clusters were found located on the chromosome and occurred already in the ancestor of *P. agardhii*/*P. rubescens*, and became partly lost during evolution of Lineage 1. In general, no increased IS element frequency in the vicinity of peptide synthesis gene clusters was observed. We found a higher proportion of IS elements in ten breaking regions related to chromosomal rearrangements and a tendency for colocalization of toxic/bioactive peptide synthesis gene clusters on the chromosome.

## Introduction

Cyanobacteria are an old form of life on earth, which occur in a wide range of aquatic and terrestrial habitats. They made a tremendous impact on the evolution of life on our planet because of oxygenic photosynthesis, and they are considered the ancestors of chloroplasts in plants. To date, cyanobacteria still contribute to global primary production, that is fixing a substantial amount of carbon (Garcia-Pichel et al., 2003) and nitrogen (Zehr et al., 2008). However, under favorable conditions, benthic and planktonic cyanobacteria multiply rapidly, and they may form dense harmful algal blooms (HABs) in aquatic ecosystems causing toxic effects in plants, invertebrates, and vertebrates, including humans and livestock. An increasing number of bloom-forming cyanobacterial species can produce over 1,100 distinct secondary metabolites (SM) (Dittmann et al., 2015) primarily via three types of biosynthetic machinery, namely, non-ribosomal peptide synthetases (NRPS), polyketide synthases (PKS), and ribosomally synthesized and post-translationally modified peptide (RiPPs) synthesis pathways (Arnison et al., 2013; Calteau et al., 2014; Kurmayer et al., 2016). Freshwater filamentous cyanobacteria *Planktothrix* are one of the major cyanotoxin and bioactive peptide producers, and they may serve as a niche constructor at ecosystem scale (Kurmayer et al., 2016). The species assigned to the genus *Planktothrix*, for example, *P. agardhii* and *P. rubescens*, are recorded from toxic cyanobacterial blooms frequently (Fastner et al., 1999; Willame et al., 2005). *Planktothrix* produce various bioactive peptide families, including hepatotoxic microcystins (MCs) (Christiansen et al., 2003; Briand et al., 2008; Metcalf and Codd, 2012), aeruginosins (Ishida et al., 2007; Kohler et al., 2014), anabaenopeptins (Itou et al., 1999; Christiansen et al., 2011), cyanopeptolins (Grach-Pogrebinsky et al., 2003; Rounge et al., 2007), microviridins (Shin et al., 1996; Philmus et al., 2008), prenylagaramides (Donia and Schmidt, 2011), and microginins (Rounge et al., 2009; Pancrace et al., 2017). The gene clusters for aeruginosins, anabaenopeptins, cyanopeptolins, microginins, and MCs consist of genes encoding NRPS and/or PKS that follow a stepwise synthesis pathway using either amino acids (NRPS) or acetyl-coenzyme A (PKS) as substrate. RiPPs are formed ribosomally through a precursor peptide (consisting of a leader peptide and a core peptide), which is post-translationally modified. Microviridins and prenylagaramides constitute two cyanobactin peptide families produced by *Planktothrix* via the RiPP pathway. Structural diversity of the cyanobactins is additionally achieved by post-translational modification, such as heterocyclization, oxidation, prenylation, and epimerization (Sivonen et al., 2010). Although they are quite diverse in size, composition, and arrangement, all cyanobactin biosynthesis gene clusters share genes encoding two proteases for precursor peptide cleavage and cyclization and accessory proteins for post-translational modification (Sivonen et al., 2010; Arnison et al., 2013).

Previous research has revealed that the genetic basis of MC synthesis is frequently influenced by certain insertion sequence (IS) elements (Christiansen et al., 2006, 2008; Chen et al., 2016; Pancrace et al., 2017). IS elements constitute the most number of mobile elements in prokaryotes, exhibiting a typical genetic structure with terminal inverted repeats, direct repeat target sequences, and an encoded transposase. IS elements may play a key role in genomic plasticity, that is by genomic rearrangement through homologous recombination in prokaryotes (Schneider et al., 2000; Zhou et al., 2008; Koonin and Wolf, 2010; Larsson et al., 2011). IS elements mutate the DNA sequence by four mechanisms during cell cycle amplification or other selective stress-induced processes: cut/paste, copy/paste, peel/paste, or co-integrate. IS elements can influence cyanobacterial SM biosynthesis gene clusters through insertion and subsequent deletion (Christiansen et al., 2006; Entfellner et al., 2017). Christiansen et al. (2008) and Chen et al. (2016) reported that non-toxic strains of *Planktothrix* frequently resulted from the (partial) deletion or inactivation of the MC synthesis gene cluster (*mcy*) through IS elements. It has been reported earlier that IS elements tend to cluster in the host genome (Zhou et al., 2008). Thus, IS elements located in the vicinity of a specific SM synthesis gene cluster might cause its inactivation, resulting in the loss or modification of bioactive peptide production. The comparison of complete genomes can reveal the potential physical relationship between IS elements and SM synthesis gene clusters. However, to date, for *Planktothrix* spp. only three strains of complete genomes, namely, *P. agardhii* NIVA-CYA126/8, PCC7805, and NIES-204, have been elucidated (Christiansen et al., 2014; Pancrace et al., 2017; Shimura et al., 2021). In addition, investigating the impact of IS elements on the population level is important to understand the influence of IS elements on a genomic scale. Therefore, in this study, we combined genomics and population genetics to characterize the distribution of IS elements and SM synthesis gene clusters within the taxonomically well-defined HAB-forming genus *Planktothrix*. Hence, we completed the genomes of 13 *Planktothrix* spp. strains assigned to three major phylogenetic lineages by using the reference genome (Christiansen et al., 2014). The phylogenetic lineages have been established using 125 *Planktothrix* strains originating from 40 water bodies located in 17 countries on three different continents (Europe, North America, and Africa) (Kurmayer et al., 2015; Entfellner et al., 2017). We focused on (i) the relationship between SM biosynthesis gene clusters and IS element distribution, (ii) the IS element-induced variation and innovation in cyanobacterial SM production, and (iii) the evolution of SM synthesis gene clusters as a result of recombination events. In particular, we analyzed seven SM biosynthesis gene clusters among 13 *Planktothrix* strains and detected 1,622 IS element copies in all the genomes. Notably, our results indicate the occurrence of IS elements with higher proportion within breaking regions related to chromosomal rearrangements resulting in colocalization of toxic/bioactive peptide gene clusters on the chromosome.

## Materials and Methods

### *Planktothrix* Strains and DNA Isolation

The genus *Planktothrix* is a monophyletic genus differentiated from other related filamentous cyanobacteria of the family Microcoleaceae of the order Oscillatoriales by genetic and morphological characters linked to its planktonic lifeform (Komárek, 2016). Eleven strains have been either assigned to *P. agardhii* (Gomont) Anagnostidis and Komárek (1988) or *P. rubescens* (DeCandolle ex Gomont) Anagnostidis and Komárek (1988) as proposed by Suda et al. (2002), forming basically two phylogenetic Lineages 1 and 2 (Kurmayer et al., 2015). Two strains were assigned to more distantly related species *P. pseudagardhii* (Suda et al., 2002), that is Lineage 3 (Kurmayer et al., 2016) or *P. tepida* (Gaget et al., 2015). *Oscillatoria* strain PCC6506, which has been reassigned to *Kamptonema* (Strunecky et al., 2014), was used as an outgroup for phylogenomic analysis.

In this study all clonal strains used for genome sequencing were purified axenic following repeated isolation on agar and grown in BG11 medium (Rippka, 1988) under sterile low-light conditions (5–10 μmol m^–2^ s^–1^, 16/8 h light–dark cycle, 15 or 23°C). The bacteria-free growth condition was confirmed regularly using DAPI staining in accordance with the standard procedure (Porter and Feig, 1980). For 13 *Planktothrix* strains assigned to the three major phylogenetic lineages, namely, Lineage 1 (No2A, No66, NIVA-CYA126/8, No365, No976, PCC7805, and PCC7811), Lineage 2 (No82, No108, No758, and PCC7821), and Lineage 3 (No713 and PCC9214), and *Kamptonema* PCC6506 axenic biomass were harvested by centrifugation and stored at −20°C. High-molecular-weight (HMW) DNA extraction was performed using the Genomic-tip 100/G kit (Qiagen, Hilden, Germany) according to the manufacturer’s protocol, i.e., following the sample preparation and lysis protocol for bacteria. For each strain, 1 g of frozen biomass was ground in liquid nitrogen and resuspended using 3.5 ml of the buffer B1 (Qiagen, contains 18.61 g/L Na_2_EDTA.2H_2_O and 6.06 g/L Tris base, 5% (v/v) of 10% Tween-20, 5% (v/v) of 10% Triton X-100, pH = 8.0). RNase A (final concentration 0.2 mg/ml), lysozyme (final concentration 5 mg/ml) and Proteinase K (final concentration 2.5 mg/ml) were added and the DNA extraction was incubated at 37°C (>30 min). 1.2 ml of buffer B2 (286.59 g/L guanidine HCL, 20% (v/v) Tween-20) were added and incubated at 60°C (>30 min). Following centrifugation (14,000g, 10 min) the clear supernatant containing HMW DNA was purified by ion exchange column purification resulting in 130–1,100 or 120–412 ng/μl of DNA as recorded in the Nanodrop spectrophotometer (ND 1000, Thermo Fisher Scientific, Vienna, Austria) or via Qubit 2.0 (Thermo Fisher Scientific), respectively.

### *Planktothrix* Genome Sequencing and Assembly

Genomes were sequenced using PacBio RS II platforms (GATC Biotech, Constance, Germany) in 2014 and/or PacBio Sequel Systems (Novogene, Beijing, China) in 2019. For PacBio RS II platforms, three SMRT cells per strain were used, whereas for PacBio Sequel Systems, only one SMRT cell per strain was used. Libraries were prepared using the standard PacBio 10-kb protocol and sequenced on a PacBio RS II system with P6-C4 chemistry (in 2014) and on the Sequel System with Sequel Sequencing Kit 3.0 (in 2019). Raw reads were filtered and assembled using the hierarchical genome assembly process (HGAP) v3 (Chin et al., 2013) for PacBio RS II platform and using HGAP v4 and Canu v2.0 (Koren et al., 2017) for PacBio Sequel System. In brief, consensus sequences were obtained using circular consensus sequencing command^1^ and converted to FASTA format using bamtools (Barnett et al., 2011; Wenger et al., 2019). Consensus sequences were assembled into contigs using Canu under “-pacbio-hifi” mode. Coverages ranged between 17 and 455-fold for the Canu and HGAP assembly reads (Supplementary Additional File 1: Table S1).

### Chromosome Completion and Plasmid Confirmation

For a number of strains (NIVA-CYA126/8, No66, No82, No108, No365, No758, PCC7805, PCC7821, and PCC9214), chromosomes and plasmids were identified on the basis of PacBio RS II assembly. Contig alignments or missing sequences between contigs were obtained by PCR using specific primers and Sanger sequencing of forward and reverse sequences (Supplementary Additional File 1: Table S2) or by re-sequencing via PacBio Sequel Systems in 2019 (No66, No108, and No365). For the other strains (No2A, No713, No976, and PCC7811), complete chromosomes and plasmids were directly obtained through the PacBio Sequel Systems. The majority of plasmids were physically confirmed by overlapping long-distance PCR, showing that a specific DNA molecule was circled as all of the primer sites could be shown to reveal PCR products (Supplementary Additional File 1: Table S2 and Supplementary Additional File 2). PCRs were performed using Phusion High-Fidelity DNA Polymerase (Thermo Scientific) according to the manufacturer’s protocol (HF Buffer, 500 nM of each primer, 200 μM of each deoxynucleotide triphosphate, 0.1 U polymerase, and 10 ng of HMW DNA). The size of PCR products was determined by gel electrophoresis (0.8% agarose gels in 0.5 × Tris-borate-EDTA buffer) and visualized using Midori Green. PCR products were either extracted from agarose gels or purified using a commercial PCR purification kit (QIAquick, Gel Extraction Kit, Qiagen) and directly sequenced (Eurofins Genomics, Ebersberg, Germany). Thirteen complete genome sequences have been submitted to EMBL-EBI under STUDY_ID PRJEB40445 (ERP124090).

### Genome Annotation and Phylogenetic Analysis

Automated genome annotations and gene predictions were performed using the genome annotation tool GenDB 2.4 (Meyer et al., 2003) as compared with the reference genome of *P. agardhii* NIVA-CYA126/8 (Christiansen et al., 2014) and reference genomes from *Arthrospira platensis* C16 (NZ_CM001632.1), *Lyngbya aestuarii* BL-J (NZ_AUZM00000000.1), and *Trichodesmium erythraeum* (JAGGDU00000000.1) or if no homolog was found via the SwissProt database. Genomic comparison was performed using the online platform EDGAR 3.0 (Blom et al., 2009) regarding the core and pan genome, average nucleotide identity (ANI), and average amino acid identity (AAI) as well as a phylogenetic tree calculated from the core genes using *Kamptonema* sp. PCC6506 as an outgroup. The phylogenetic tree was calculated using the FastTree software^2^ to generate approximately-maximum-likelihood phylogenetic trees and Shimodaira-Hasegawa local support values (Shimodaira and Hasegawa, 1999). All genes were compared pairwise within chromosomes or plasmids using 97% identity cutoff and >90% query coverage to quantify paralogs. In order to quantify gene functions the Clusters of Orthologous Groups (COG) database was used as transferred from the NIVA-CYA126/8 reference genome (Christiansen et al., 2014).

### Insertion Sequence Element Assignment

Insertion sequence element transposases were assigned or predicted using ISsaga 2.0 (Varani et al., 2011), available through the ISfinder database (Siguier et al., 2006; date August 4th 2020). The IS elements annotated by GenDB were matched with those identified by ISfinder and BlastX with an e value of <10^–20^. Considering that terminal repeats of ISs are often small and poorly characterized and in many cases do not allow a precise definition, the predicted ORF of a specific IS element was used for further analysis. Copy numbers of different IS elements were calculated from all 13 sequenced genomes by BlastN using a threshold e value of 1e^–20^. We used ORF length, non-interrupted translation, and copy number as criteria, that is shorter sequences occurring in higher copy number only were considered as fragments, to differentiate full-length transposases from fragments.

### Secondary Metabolite Biosynthesis Genes

Seven previously described SM biosynthesis gene clusters were analyzed among the 13 *Planktothrix* strains: microcystin (*mcyT-J*) biosynthesis (Christiansen et al., 2003), aeruginosin (*aerA-N*) biosynthesis (Ishida et al., 2007), anabaenopeptin (*apnA-E*) biosynthesis (Christiansen et al., 2011), cyanopeptolin (*ociD-C*) biosynthesis (Tooming-Klunderud et al., 2007), microginin (*micA-E*) biosynthesis (Rounge et al., 2009), microviridin (*mvdA-F*) biosynthesis (Philmus et al., 2008), and prenylagaramide (*pagC-G*) biosynthesis (Donia and Schmidt, 2011). In addition, the automatic annotation revealed some unknown NRPS/PKS genes occurring in strains of Lineage 3. Net distances between SM synthesis gene clusters (or fragments) and individual IS elements were calculated and statistically compared with normal distribution using the Kolmogorov-Smirnov test (Sigma Plot 14.0).

### Secondary Metabolite Peptides

In order to investigate peptide synthesis all strains were incubated in liquid BG11 medium (Rippka, 1988) at 15°C (*P. agardhii, P. rubescens*) or 23°C (*P. pseudagardhii, P. tepida*) under continuous low light conditions (10 μmol m^–2^ s^–1^, Osram Type L30W/77 Fluora) and harvested during early logarithmic growth phase (usually after 14 days). Harvested cells on glass fiber filters were dried, and 2–14 mg (median 4.8) of dry weight were extracted in 50% (v/v) aqueous methanol as described previously by shaking on ice (Kosol et al., 2009). Peptides were separated by HPLC (HP 1100, Agilent, Vienna, Austria) using a water/acetonitrile (0.05% trifluoroacetic acid) gradient from 80:20 to 50:50 in 45 min at a flow rate of 1 mL min^–1^ and at 30°C oven temperature via a LiChrospher 100 RP18e octadecylsilica (5 μm particle size) sorbent packed in a LiChroCART 250-4 cartridge system (Merck, Darmstadt, Germany) (Kosol et al., 2009). An electrospray ionization mass spectrometer ion trap (amaZonSL, Bruker, Vienna, Austria) was coupled to the HPLC as described (Entfellner et al., 2017). Peptides were detected in positive-ion mode using nitrogen as sheath gas (43 psi, 8 L min^–1^, 300°C) and helium as auxiliary gas. The capillary voltage was set to 5 kV. Mass screening and automated fragmentation were performed within one run using the two precursor masses with high intensity for MS^2^ fragmentation and one for MS^3^ fragmentation. Peptides were determined on the basis of the retention time, mass, and fragmentation pattern and assigned to the peptide families based on the fragmentation pattern or the predicted mass of a precursor peptide.

### Graphical Tools

SnapGene was used to visualize primer positions on plasmids. Synteny plots between strains were calculated using R2cat (Husemann and Stoye, 2010). BioCircos package in RStudio (Cui et al., 2016) was used to visualize the position of SM synthesis gene clusters, breaking regions of chromosomal rearrangements, and individual IS element groups.

## Results

### Comparison of *Planktothrix* Genomes

**FIGURE 1:**
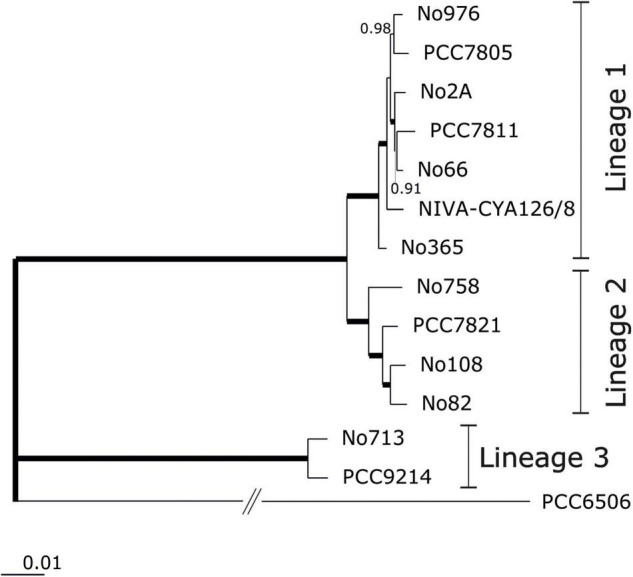
Phylogenomic tree calculated from 2,881 core genes using the FastTree software (http://www.microbesonline.org/fasttree) to generate approximately-maximum-likelihood phylogenetic trees (EDGAR 3.0) and Shimodaira-Hasegawa local support values (Shimodaira and Hasegawa, 1999). Branches showing local support of 1.0 are indicated with thick lines. Assignment of strains to phylogenetic lineages as described by Entfellner et al. (2017). *Kamptonema* (*Oscillatoria*) strain PCC6506 was used as an outgroup [for corresponding average amino acid identities (AAI) or nucleotide identities (ANI) see Supplementary Additional File 3: Figure S2].

We completed the genome of 13 *Planktothrix* strains, which were previously assigned to three different phylogenetic lineages (Entfellner et al., 2017). For each strain, a single circular chromosome and a variable number of plasmids (0–5) were identified (Table 1). For strain NIVA-CYA126/8 the previously published genome (Christiansen et al., 2014) was re-sequenced and closed. For strain PCC7805 the genome was published earlier by Pancrace et al. (2017), however, has been resequenced during this study. For both strains the resequenced total genome sizes were found 50 kbp increased (1.1% of genome size). For NIVA-CYA126/8 the larger plasmids (119, 90, and 52 kbp) were confirmed while the two smaller plasmids (5 kbp) were found integrated into the 90 kbp plasmid. For PCC7805 the megaplasmid was confirmed while the 5 kbp plasmid was not found. Within *Planktothrix* spp. the chromosome size varied between 4.72 Mbp (i.e., strain No2A) and 6.8 Mbp (PCC 9214), resulting in 4,305–5,796 predicted protein-coding genes (ORFs). *Planktothrix agardhii* strains of Lineage 1 (1A) had the smallest chromosomes; *P. rubescens* or *P. agardhii* strains of Lineage 2 had larger chromosomes, whereas those of Lineage 3 had the largest chromosomes. All strains had the same number of rRNA copies (three 5S, four 16S, and four 23S). However, tRNAs varied from 42 among strains of Lineages 1 and 2 up to 48 (PCC9214) or 56 (No713) in Lineage 3. In 13 chromosomes, we obtained 2,881 core genes and 9,284 pan genes (Supplementary Additional File 3: Figure S1A). The core genome development curve reached a stable minimum, indicating that the number of core genes will not decrease when *Planktothrix* spp. strains were further considered. For the pan genome, we obtained a saturation curve with a slight increase; therefore, we could obtain additional gene information when further strains assigned to *Planktothrix* spp. were considered. Considering closely related *P. agardhii*/*P. rubescens* strains, not only reduced variability but still no saturation was reached for the pan genome (Supplementary Additional File 3: Figure S1B). When calculated for each lineage, again no saturation for pan genes was observed (Supplementary Additional File 3: Figure S1C). Instead pan genes increased distinctly between Lineages 1 and 2 and Lineages 2 and 3 by 34–41 and 52–54%, respectively (Supplementary Additional File 3: Figures S1C,D). The phylogenomic tree calculated from core genes (Figure 1) revealed that Lineages 1 and 2 are sister lineages, to the exclusion of basal Lineage 3. In agreement the AAI and ANI matrices revealed that strains of Lineages 1 and 2 were found most closely related, whereas strains of Lineage 3 (No713 and PCC9214) were found more distinct (Supplementary Additional File 3: Figure S2). Lineage 1 differed from Lineage 2 between 97.8 and 98.3% (AAI) or 95.3–96.3% (ANI) which is close to the cutoff frequently used for species demarcation (Jain et al., 2018).

**TABLE 1:** General characteristics of 13 *Planktothrix* spp. strains and genomes as well as origin and year of isolation (if known).

Taxonomic affiliation	Strain	Place and year of isolation	Phyl. lineage	Total genome size (Mb)	Chr. size (Mb)	Plasmid size (Kb)^3^	GC cont. (%)	No. of genes	5/16/23S rRNAs; tRNAs	Total IS copy number	% of ISs (Chr.)	% of ISs (Plasmid)	Genebank assembly access no.
*Planktothrix agardhii*	NIVA-CYA 126/8	L. Langsjön (FI) 1984	1	5.10	4.84^1^	51.8; 90.5; 119.6	38.8	4494	3/4/4; 42	105	1.6	3.5; 6.5; 7.5	GCA_904830765
*Planktothrix agardhii*	No2A	L. Markusbölefjärden (FI), unknown	1	4.79	4.72	7.3; 62.3	38.4	4305	3/4/4; 42	75	1.3	0.0; 6.8	GCA_904830775
*Planktothrix agardhii*	No66	Jägerteich (AT) 2001	1	4.95	4.74	44.1; 70.6; 94.1	39.8	4434	3/4/4; 42	81	1.2	6.7; 16.4; 0.0	GCA_904830855
*Planktothrix agardhii*	No976	Ft. Lowell, Tucson (US) 2009	1	4.91	4.75	5.3; 15.8; 36.1; 37.8; 61.1	38.9	4423	3/4/4; 42	67	0.8	0.0; 0.0; 0.0; 10.0; 13.7	GCA_904830935
*Planktothrix agardhii*	PCC7805	Veluwemeer (NL) 1972	1	4.90	4.75^2^	153.0	39.7	4446	3/4/4; 42	88	1.3	6.0	GCA_904830915
*Planktothrix agardhii*	PCC7811	Paris, Vert le Petit (FR) 1964	1	5.16	4.80	39.0; 48.5; 77.6; 88.9; 102.9	37.0	4645	3/4/4; 42	88	1.4	0.0; 0.0; 2.8; 1.3; 4.3	GCA_904830885
*Planktothrix agardhii*	No365	Moose L. (CA) 2006	1A	4.85	4.71	23.0; 39.7; 64.5; 136.0	39.0	4482	3/4/4; 42	79	1.1	3.7; 0.0; 14.7; 0.7	GCA_904830845
*Planktothrix rubescens*	No82	Ammersee (DE) 2001	2	5.69	5.45	14.7; 68.6; 153.4	39.3	5043	3/4/4; 42	137	2.1	15.6; 5.8; 0.9	GCA_904848565
*Planktothrix rubescens*	No108	Irrsee (AT) 2001	2	5.58	5.41	63.1; 109.0	39.0	4970	3/4/4; 42	128	1.9	5.0; 7.7	GCA_904830785
*Planktothrix rubescens*	PCC7821	L. Gjersjoen (NO) 1971	2	5.69	5.46	13.0; 48.5; 78.6; 94.0	39.5	5104	3/4/4; 42	165	2.6	0.0; 2.7; 4.4; 5.9	GCA_904830895
*Planktothrix agardhii*	No758	L. Hormajärvi (FI) 2007	2A	5.64	5.64	–	39.5	4915	3/4/4; 43	145	2.5	n/a	GCA_904830945
*Planktothrix pseudagardhii*	No713	L. Saka (UG) 2007	3	6.65	6.60	53.0	39.7	5696	3/4/4; 56	282	3.7	4.3	GCA_904830925
*Planktothrix tepida*	PCC9214	Banguis Landjia (CF) 1989	3	6.80	6.27	5.9; 49.9; 83.7; 386.0	37.6	5796	3/4/4; 48	182	2.1	0.0; 6.0; 3.6; 2.6	GCA_904830955

*Taxonomic affiliation of P. agardhii, P. rubescens and P. pseudagardhii according to Suda et al. (2002) and of P. tepida (Gaget et al., 2015). Assignment of strains to phylogenetic lineages described by Entfellner et al. (2017).*

*^1^Resequencing (de novo) and genome closing of previously published genome (Christiansen et al., 2014).*

*^2^Identical strain for which genome was published previously, Access. No. LO018304 (Pancrace et al., 2017).*

*^3^Underlining indicates plasmids confirmed by PCR analysis; for strain NIVA-CYA126/8 plasmids were confirmed previously (Christiansen et al., 2014).*

We used synteny plots to investigate chromosomal gene arrangement and explore differences in genomic structure among strains. We found a similar chromosomal structure among strains within a lineage and only a few chromosomal rearrangements among Lineages 1, 1A, 2, and 2A. By contrast, the chromosomal arrangement of *P. agardhii*/*P. rubescens* strains (Lineages 1 and 2) compared with strains of Lineage 3 (No713 and PCC9214) was completely different but rather indifferent between the two strains No713 and PCC9214 (Supplementary Additional File 3: Figure S3).

Paralogous genes were calculated to identify the factors contributing to chromosome size variation, which contributed only 1.0% (strain No976) to 4.2% (strain No713) of all genes (Table 2). Paralogs comprised IS elements ranging from 15.7% (strain No66) to 82.3% (strain No713). The other duplicated genes were assigned to photosystem II, gas vesicle proteins, reverse transcriptases, and unknown proteins (Supplementary Additional File 1: Table S3). Thus, on a nucleotide basis, the percentage of duplicated genes (without transposases) was <1%. Therefore, apart from a relatively minor influence of IS elements, duplication of genes was also of relative minor importance for chromosome size increase among *Planktothrix* spp. Thus, the major part of chromosome size variation must have resulted from gene loss processes (ancestor of Lineages 1 and 2) and horizontal gene transfer (HGT).

**TABLE 2:** Paralogous genes identified from *Planktothrix* spp. chromosomes (left) and deviating genes compared to the reference NIVA-CYA126/8 (right).

		Paralogous genes						Genes not present in reference NIVA-CYA126/8^a^			
	
*Planktothrix* strain	Lineage	Number of paralogs	% CDS of chrom.	% tpn of paralogs	Number of paralogs (non-tpn)	% CDS of chrom. (non-tpn)	% unchar. hyp. prot.	Number of genes	% CDS of chrom.	% unchar. hyp. prot.	Nucleotides (bp)
NIVA-CYA126/8	1	71	1.7	46.5	38	0.9	18.4	123	0.6	83.7	26652
No2A	1	65	1.5	38.5	40	0.9	7.5	374	6.9	64.2	279681
No66	1	134	3.1	15.7	113	2.6	55.8	436	6.9	69.3	280236
No976	1	44	1.0	18.2	36	0.8	2.8	454	7.6	65.2	307398
PCC7805	1	60	1.4	41.7	35	0.8	5.7	448	7.4	64.5	303768
PCC7811	1	72	1.7	22.2	56	1.3	12.5	414	7.2	64.3	297504
No365	1A	63	1.5	33.3	42	1.0	28.6	429	7.1	62.9	285246
No82	2	125	2.6	52.0	60	1.2	25.0	1011	17.1	54.9	802836
No108	2	126	2.6	46.0	68	1.4	16.2	1039	17.6	55.2	822132
PCC7821	2	170	3.5	55.3	76	1.6	28.9	1042	17.0	57.8	798441
No758	2A	145	3.0	55.2	65	1.3	23.1	1173	20.4	53.1	995394
No713	3	237	4.2	82.3	42	0.9	28.6	2435	40.7	46.9	2301945
PCC9214	3	186	3.5	55.9	82	1.5	7.3	2119	37.5	46.0	2008611

*^a^Genbank acc. no. NZ_CM002803.1; CDS, coding DNA sequence; tpn, transposases.*

Based on synteny analysis, many deviating genes appeared singular. We also found some larger regions encoding putatively functional gene clusters. Two of these operons were described earlier as the phycoerythrin gene cluster among red-pigmented *P. rubescens*/*P. agardhii* strains (Tooming-Klunderud et al., 2013) or the nitrogen-fixation gene cluster described for strain PCC9214 in Lineage 3 (Pancrace et al., 2017). When compared with the NIVA-CYA126/8 reference genome, strains among Lineage 1 slightly differed in deviating genes by 6.9–7.6% only. Strains among Lineage 2 differed by 17.0–20.4%, whereas strains among Lineage 3 differed by 37.5–40.7%. Based on the phylogenomic tree described above (Figure 1), the strains of Lineage 2 gained 14–20% of their genomic information possibly through HGT, whereas ancestors of Lineages 1 and 2 possibly lost up to 40.7% from more basal genotypes of Lineage 3. Correspondingly Venn diagrams revealed a higher overlap for both core and pan genes between lineages 1 and 2, while the overlap with Lineage 3 was smaller (Supplementary Additional File 3: Figure S4). In order to find out whether for disjunct core and pan genes certain gene functions occurred more frequently the COGs were assigned. It should be noted that only 20–43% and 20–34% for core and pan genes, respectively were assigned to COG categories. Overall for core genes the COG categories showed a rather similar proportion when compared between lineages. Lineage 3 showed higher percentage of COGs related to primary metabolism (i.e., C, G, E, H, and I). For pan genes, notably COG X (Mobilome: prophages, transposons including transposases) occurred most frequently among Lineage 2.

### Insertion Sequence Elements

**FIGURE 2:**
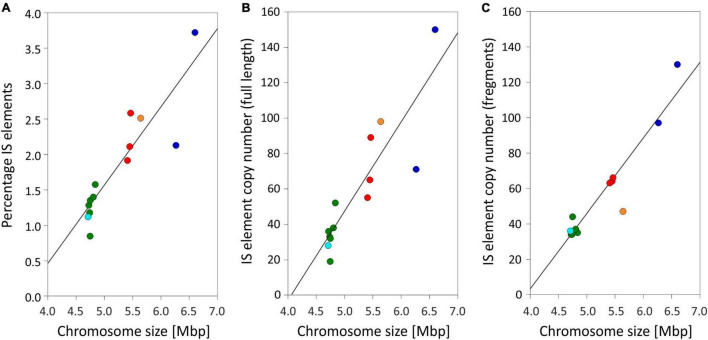
Relationship between chromosome size and **(A)** percentage of IS elements on a nucleotide basis (R^2^ = 0.81); **(B)** number of full-length IS element copies per chromosome (R^2^ = 0.79); **(C)** number of IS element fragments per chromosome (R^2^ = 0.9). Symbols: Green (phylogenetic Lineage 1), turquoise (Lineage 1A), red (Lineage 2), orange (Lineage 2A), and blue (Lineage 3).

Using the completed genomes, we performed an exhaustive comparison of the composition and localization of IS elements. In total, we found 1,622 IS element copies among the 13 genomes (Supplementary Additional File 1: Table S4). Thereof 865 (53.7%) full-length transposases of IS elements were found and classified as putatively active. The IS element copy number per strain varied between 68 (No976) and 273 (No713). In the chromosomes, IS element transposases made up 0.8–3.7% (Table 1) on nucleotide basis, which increased with the increase of chromosome size (Figure 2). Similarly the copy number of full-length transposases and fragments thereof increased with chromosome size. The percentage of IS elements on plasmids was more variable as some plasmids were found without any IS elements, and others were found carrying up to 16.4% (Table 1).

**FIGURE 3:**
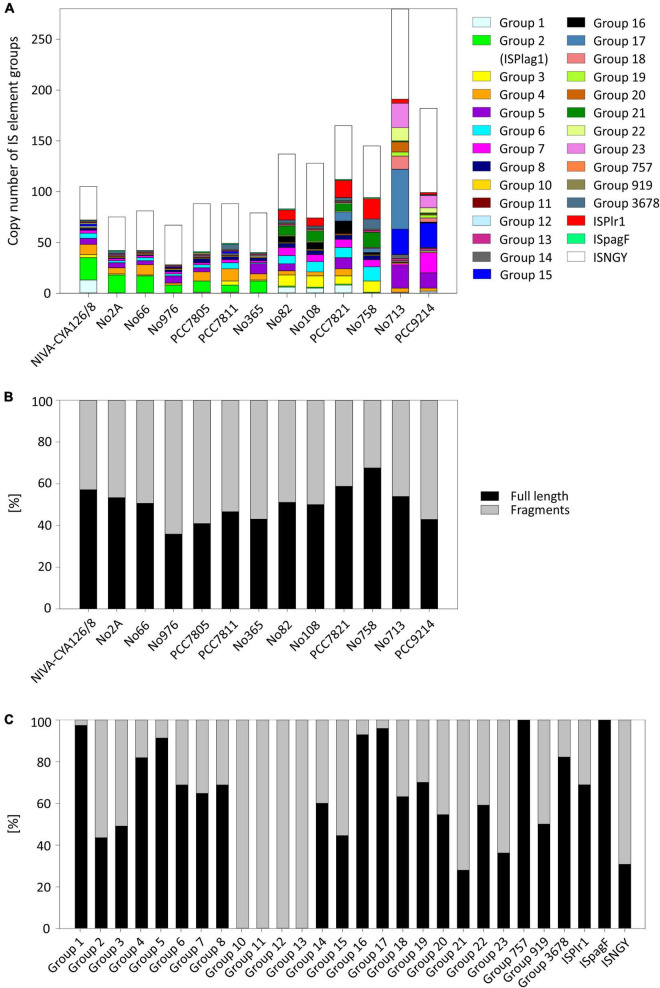
Occurrence and frequency of insertion sequence (IS) elements among 13 *Planktothrix* spp. strains. **(A)** Copy number of 27 IS element groups per genome of a strain. **(B)** Percentage of full-length IS elements vs. fragments of IS elements per genome. **(C)** Percentage of full-length IS element group vs. fragments of IS element group. NGY, no IS element group has been assigned.

We identified 15 known IS element families by using the IS finder platform. Nevertheless, 7.8% of IS element copies were not classified to IS element families yet. The most abundant IS element family was IS200/IS605 (30%). For a precise insight, we further subdivided the main part of IS elements (58.7%) into 27 groups because of blastN similarity (Figure 3A and Table 3). The majority of IS element groups occurred chromosomally only, whereas some IS element groups occurred on chromosomes and plasmids (e.g., groups 2, 4, 5, and 16). The frequency of these IS element groups and its phylogenetic distribution differed enormously: For IS element group 10, we found 13 copies in total, i.e., each strain had exactly one copy. Group 7 was also present in all strains; however, the copy number varied remarkable, as some strains had only one copy, and strain PCC9214 of Lineage 3 hosted 20 full-length copies. In addition, IS element group 5 was present in all lineages in a high copy number. IS element group 1, flanking the *mcy* gene cluster (Christiansen et al., 2003), occurred in full length in Lineages 1 and 2 in high copy number, whereas only few copies were found in Lineage 3. Opposite results were found for IS element group 15 and 23, which showed high copy numbers and occurred only in strains No713 and PCC9214 and thus appeared to be phylogenetically restricted to Lineage 3. Furthermore, with one exception IS element group 16 occurred in Lineage 2 only. IS element group 2 (ISPlag1), which is known to cause inactivation and deletion of the *mcy* gene cluster (Christiansen et al., 2008), showed the most copies (43 full-length copies and 56 fragments) and occurred mostly among strains of Lineage 1. Only fragments of ISPlag1 were found in strains of Lineage 2. ISPlr1, which is also known to inactivate the *mcy* gene cluster by insertion (Chen et al., 2016), was found frequently in Lineage 2, and few copies were found in Lineage 3. Furthermore, IS element group 4 was found adjacent to the anatoxin biosynthesis gene cluster in *Kamptonema* (*Oscillatoria*) strain PCC6506 (Mejean et al., 2009), and it occurred in full-length in Lineages 1 and 2, whereas Lineage 3 carried fragments only.

**TABLE 3:** Occurrence and frequency of 27 insertion sequence (IS) element groups among *Planktothrix* spp. genomes.

IS element group	Closest homolog by BLASTp in NCBI and ISfinder	Lineage1 and 1A (*n* = 7)			Lineage 2 and 2A (*n* = 4)			Lineage 3 (*n* = 2)		
		Number of strains	Length (bp)	Copy number	Number of strains	Length (bp)	Copy number	Numberof strains	Length (bp)	Copy number
1*^a^*	IS630, transposase *P. agardhii*	3	1074	1–13	3	1059–1074	5–8	2	1059	2 (1)
2*^b^*	IS701, transposase *P. agardhii*, ISPlag1	7	1014	1–13 (5–11)	4	–	(1)	–	–	–
3	IS200/IS605, transposase ISAsp12, *Anabaena sp.*	5	1107–1371	1–4	4	1215–1362	1-7 (4-10)	–	–	–
4*^c^*	IS1634, transposase *Planktothrix sp.*	7	1569–1644	2–10 (2–3)	3	1644	4–7	2	–	(3–4)
5	IS5, transposase, *P. agardhii*	6	921	4–10	2	921	5–9	2	921	14–19 (1–3)
6	IS200/IS605, transposase ISAsp6, *Anabaena sp.*	7	1272–1281	1–4 (1–2)	4	1272–1410	6–13 (1–4)	–	–	–
7	IS200/IS605 transposase TpnB *P. prolifica*	7	1212	1 (2)	4	1218	4–6 (1–4)	2	1227–1230	1–20 (1)
8	IS200/IS605, transposase ISMae41, *Microcystis aeruginosa*	7	1173	1–3 (1)	4	1170–1173	2–3 (1–2)	–	–	–
10	Transposase *P. tepida*	7	–	(1)	4	–	(1)	2	–	(1)
11	Transposase *P. agardhii*	4	–	(1)	1	–	(1)	1	–	(1)
12	IS200/IS605, transposase *Planktothrix sp.* UBA8407	5	–	(1)	–	–	–	2	–	(1)
13	IS630, transposase *P. tepida* PCC 9214	–	–	–	–	–	–	2	–	(2)
14	IS200/IS605, transposase *P. tepida*	–	–	–	–	–	–	2	1230	1–2 (2)
15	IS701, transposase *P. tepida*	3	–	(1–2)	–	–	–	2	1236–1335	11–13 (12–13)
16	S701, transposase *Planktothrix*	–	–	–	4	1266	1–12 (1)	1	–	(1)
17	IS630, transposase *Planktothrix*	–	–	–	2	1113	3–9 (1)	1	1113	57 (2)
18	IS5, transposase ISMae6, *Microcystis aeruginosa*	1	1497	1	1	–	(1)	2	1494	1–10 (3)
19	IS200/IS605, transposase *Planktothrix*	–	–	–	3	1203–1215	1	2	1212	2 (1–2)
20	IS4, transposase *P. mougeotii*	–	–	–	–	–	–	2	1326	6 (1-4)
21	ISAs1, transposase *Planktothrix*	–	–	–	4	1119	12 (3–10)	2	–	(1)
22	ISAzo13, transposase *P. tepida*	1	–	(1)	3	–	(1)	2	1212	4–9 (1–4)
23	IS630, transposase *P. tepida*	–	–	–	–	–	–	2	1038	3–10 (9–14)
757	IS200/IS605, transposase TnpB *P. agardhii*	7	1143	1	4	1143	1–2	–	–	–
919	IS200/IS605, transposase TnpB *Planktothrix*	5	1251	1 (1–2)	4	1251	1 (1)	–	–	–
3678	IS200/IS605, transposase TnpB *Planktothrix*	4	1173–1182	1–3 (1)	4	1143–1173	2–8 (0–2)	1	1140	1
ISPlr1*^d^*	ISAs1, transposase ISPlr1, *P. rubescens*	–	–	–	4	1092	6–17 (2–7)	2	1092	2 (2)
ISpagF	Transposase, *Planktothrix*	4	753–816	1	3	702–768	1	–	–	–

*For full-length IS element copies, the length of encoded transposases is indicated. Numbers in brackets indicate copy numbers of corresponding fragments. ^a^Flanking the mcy gene cluster. ^b^Inactivating/deleting the mcy gene cluster. ^c^Adjacent to ana gene cluster. ^d^Inactivating the mcy gene cluster.*

In general, the ratio between full-length IS elements and fragments of IS elements was balanced and strain specific but not lineage specific. Strain No976 and strain No758 had the lowest (36%) and the highest (68%) content of full-length transposases, respectively (Figure 3B). We found no general difference between chromosomes and plasmids with regard to full-length vs. fragments. With regard to the 27 IS element groups, the ratio of full-length IS elements and fragments differed remarkably. Numerous IS element groups (groups 1, 16, 17, 757, and ISpagF) contained a high proportion of full-length copies (>90%). Other IS element groups had less full-length copies; in particular, groups 10, 11, 12 and 13 occurred only as fragments (Figure 3C).

### Secondary Metabolite Synthesis Gene Cluster Distribution

**FIGURE 4:**
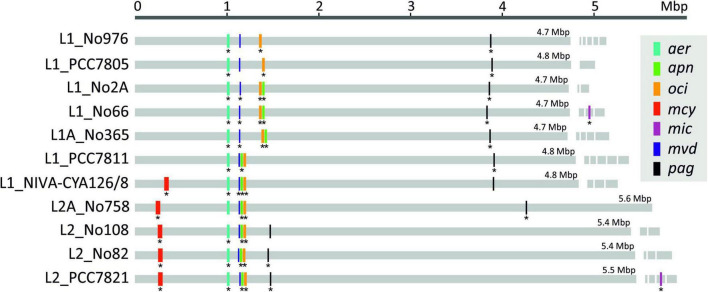
Location of seven secondary metabolites (SM) synthesis gene clusters among 11 *Planktothrix agardhii*/*P. rubescens* chromosomes and plasmids (Only the *mic* genes have been located on plasmids). The star indicates that synthesized peptide products have been detected by HPLC–MS*^n^*.

We localized seven previously elucidated SM synthesis gene clusters, including two NRPS gene clusters (*apnA-E* and *ociD-C*), two NRPS/PKS hybrid gene clusters (*aerA-N* and *mcyT-J*), two RiPP gene clusters (*mvdA-F* and *pagC-G*) on the chromosomes, and another NRPS/PKS gene cluster (*micA-E*) occurring on plasmids (Table 4 and Figure 4). Strains of Lineage 1 carried 4–6 of these SM synthesis gene clusters, whereas strains of Lineage 2 had 6–7 clusters. Four biosynthesis gene clusters (*aer*, *mvd*, *oci*, and *pag*) were present among all strains of Lineages 1 and 2. By contrast, the *apn* and *mcy* gene clusters were distributed irregularly, that is *apn* and *mcy* genes occurred irregularly among strains of Lineage 1, but they were always present among strains of Lineage 2. The *mic* gene cluster, located on plasmids, was randomly distributed, as it was only found in two strains, namely, No66 (Lineage 1) and PCC7821 (Lineage 2). By contrast, the two strains of Lineage 3 had none of these abovementioned SM synthesis gene clusters. However, we found several putative NRPS/PKS genes among strains of Lineage 3. Similarly, among strains of Lineage 1 and 2, unknown NRPS/PKS genes were found.

**TABLE 4:** Occurrence and nucleotide length (in kbp) for seven SM synthesis gene clusters among *Planktothrix* spp.

*Planktothrix* strain	Phylogenetic lineage	*aerA-N*	*apnA-E*	*ociD-C*	*mcyT-J*	*micA-E*	*mvdA-F*	*pagC-G*
NIVA-CYA126/8	1	30.0	23.9	28.0	53.2	–	5.1	13.1
No2A	1	26.8	23.9	31.2	–^1^	–	5.2	15.8 (1)
No66	1	26.8	23.9	31.2	–^1^	21.1 (1)	5.2	15.3
No976	1	26.5	–^1^	32.6	–^1^	–	5.6	11.4
PCC7805	1	27.2	–^1^	32.6	–^1^	–	4.8	13.4 (1)
PCC7811	1	26.8	23.9	28.8	–^1^	–	4.8	12.0 (1)
No365	1A	30.7	24.0	32.9	–^1^	–	5.6	13.5 (1)
No82	2	26.6	24.0	31.2	52.0	–	4.8	12.5 (1)
No108	2	23.5	23.9	32.6	52.0	–	4.8	9.5
PCC7821	2	26.6	23.9	33.2	52.0	17.1 (1)	5.6	13.9 (1)
No758	2A	23.9^2^	23.9	32.8	53.0	–	5.1	12.8 (1)
No713	3	–	–	–	–	–	–	–
PCC9214	3	–	–	–	–	–	–	–

*The number of IS elements located within a SM synthesis gene clusters is indicated in parentheses (see also Supplementary Additional File 3: Figure S5).aer, aeruginosin synthesis (NRPS/PKS); apn, anabaenopeptin synthesis (NRPS); oci, cyanopeptolin synthesis (NRPS); mcy, microcystin synthesis (NRPS, PKS); mic, microginin synthesis (NRPS/PKS); mvd, microviridin synthesis (RiPP); pag, prenylagaramide synthesis (RiPP). ^1^Fragments (remnants) only. ^2^Partial deletion.*

Figure 4 shows the distribution of SM synthesis gene clusters on the chromosomes. Notably, individual gene cluster positions changed in congruence with the phylogeny of the strains. Strains of Lineage 1 (e.g., No66) were found to have the *apn* and *oci* gene clusters distant to the *mvd* gene cluster, whereas among all strains of Lineage 2 and some strains of Lineage 1 (e.g., NIVA-CYA126/8) the *apn* and *oci* genes were found rather close to the *mvd* genes, i.e., forming a so-called meta gene cluster of *mvd*–*apn*–*oci*. Similar evolution can be observed for the *pag* gene cluster, which was located far away from all other SM synthesis gene clusters among strains of Lineages 1, 1A, and 2A, but *pagC-G* genes became localized closer to the meta gene cluster among strains of Lineage 2.

### Secondary Metabolite Synthesis Gene Cluster Functionality

In general, whenever we found one of the abovementioned NRPS or NRPS/PKS gene cluster in the genome, we also detected corresponding putative peptides, confirming a high share of SM synthesis gene cluster functionality in *Planktothrix* (Table 5, Supplementary Additional File 1: Tables S5, S6 and Supplementary Additional File 3: Figure S5). In addition, for the *mic* gene cluster carried on plasmids (No66, PCC7821), the produced microginins were detected. However, an inactive *aer* gene cluster of strain No758 occurred because of partial deletion as well as a putatively inactive *oci* gene cluster of strain PCC7811. In contrast to NRPS, the two RiPP gene clusters were found frequently inactive. Within the *mvd* gene cluster, strains were found to carry one or two potential precursor peptide genes (*mvdE* and *mvdF*). For strains carrying *mvdE* (No66, No2A, No365, and NIVA-CYA126/8), the predicted peptides were detected. Strain No365 had two slightly different *mvdE* precursor peptide genes resulting in two different microviridin structural variants. On the contrary, for strains carrying only the *mvdF* precursor peptide gene, the corresponding peptide could not be detected. The *pag* gene clusters in *Planktothrix* showed a variable number of precursor peptide genes (*pagE*) ranging from one *pagE* copy in strain No108 to 12 *pagE* copies in strain No66 (Supplementary Additional File 3: Figure S5), indicating gene duplication and potential structural diversification. Some strains seemed to carry several active *pagE* genes (e.g., three in No976), whereas other strains had no active *pagE* (e.g., NIVA-CYA126/8). Consistent with Donia and Schmidt (2011), both genes encoding prenylagaramide B 1 (*pagE6*) and C 2 (*pagE7*) were detected in *P. agardhii*. It is interesting to note that active *pagE* genes often were found to contain a core motif GLTPH/L (Supplementary Additional File 1: Table S7) which has been suggested as a restriction site for the N-terminal protease A previously (Gu et al., 2018). Moreover, some strains of *P. rubescens* were reported to produce one or two variants of planktocyclin (Baumann et al., 2007; Kurmayer et al., 2015). Using backtranslation of the amino acid sequence constituting planktocyclin Pro-Gly-Leu-Val-Met-Phe-Gly-Val (resulting in 12,288 possible nucleotide sequences of 24 bp in length), only one exact BLASTn hit was found in the genome of strain No66. This exact match formed part of the precursor genes *pagE* described for the *pag* gene cluster previously (Donia and Schmidt, 2011). Therefore, we concluded that the *pag* gene cluster corresponded to the synthesis of prenylagaramide and planktocylin (lacking prenylation). Furthermore, we found a correlation between the presence of prenylated peptides and prenyltransferases (*pagF*). The strains PCC7805, PCC7811, No365, and No758 carried a highly similar *pagF* gene, and these strains also produced *O*-prenylated tyrosine-containing peptides (e.g., Pag B 1 and C 2). For strains No2A and No82 (and also in the planktocyclin-inactive strain NIVA-CYA126/8), we detected a lower similarity of the *pagF* gene (Supplementary Additional File 3: Figure S6). Recently, Shimura et al. (2021) reported dissimilarity of the *pagF* gene using strain PCC7821, resulting in C-prenylation of tryptophan, which has been previously known as oscillatorin (Sano and Kaya, 1996). Other strains (No66 and No976) lacking prenyltransferase produced planktocyclins but no prenylagaramides.

**TABLE 5:** Peptide products resulting from seven SM synthesis pathways among 13 genome-sequenced *Planktothrix* spp. strains.

*Planktothrix* strain	Aeruginosins	Anabaenopeptins	Cyanopeptolins	Microcystins	Microginins	Microviridins	Planktocyclins/Prenylagaramides
NIVA-CYA126/8	Aer 126A, put. Aer 730	AP 908, AP 915	Cpt 960	[D-Asp^3^]MC-LR, [D-Asp^3^]MC-RR	n/a	Mvd K	n.d.
No2A	put. Aer 714, 716	AP B	put. Cpt 1153	n/a	n/a	Mvd I	put. Pla 1060
No66	put. Aer 714, 716	AP B	put. Cpt 1153	n/a	put. Mic 548, 582, 616	Mvd I	Planktocyclin
No976	put. Aer 820, 854, 868	n/a	put. Cpt 993	n/a	n/a	n.d.	put. Pla 992, 1580, 1827
PCC7805	put. Aer 740, 754, 774, 788, 834, 868	n/a	put. Cpt 993, 1088	n/a	n/a	n.d.	Pag B, Pag C
PCC7811	put. Aer 714, 716	AP F, Oscillamide Y	n.d.	n/a	n/a	n.d.	Pag B
No365	put. Aer 868, 892	AP A, AP B	put. Cpt 1136	n/a	n/a	put. Mvd 1640, 1803	put. Pag 1064
No82	put. Aer 592, 616, 632	AP F, Oscillamide Y	Oscillapeptin J	[D-Asp^3^]MC-LR, [D-Asp^3^]MC-RR	n/a	n.d.	put. Pla 1125
No108	put. Aer 770, 804	AP B, AP C, AP F put. AP 822, 836	put. Cpt 1074	[D-Asp^3^]MC-LR, [D-Asp^3^]MC-RR	n/a	n.d.	n.d.
PCC7821	put. Aer 592, 616, 632	AP F, Oscillamide Y	Oscillapeptin G Frag. Osc. G	[D-Asp^3^]MC-LR, [D-Asp^3^]MC-RR	Oscillaginin A, Oscillaginin B	n.d.	Oscillatorin, put. Pag 1969
No758	n.d.	AP B, AP C	put. Cpt 1003, 1037, 1049, 1083	put. [D-Asp^3^]MC-RY, [D-Asp^3^]MC-RR	n/a	n.d.	put. Pla 839, put. Pag 1501
No713	n/a	n/a	n/a	n/a	n/a	n/a	n/a
PCC9214	n/a	n/a	n/a	n/a	n/a	n/a	n/a

*The raw data of HPLC-MS analysis as well as a list of protonated masses and assigned peptides for all 13 strains have been included in Supplementary Additional File 1: Tables S5, S6. n/a, not applicable; n.d., not detected; put., putative; Aer, Aeruginosin; AP, Anabaenopeptin; Cpt, Cyanopeptolin; MC, Microcystin; Mic, Microginin; Mvd, Microviridin; Pag, Prenylagaramide; Pla, Planktocyclin.*

### Relationship Between Insertion Sequence Elements and Secondary Metabolites Synthesis Gene Clusters

We calculated net distances between the location of SM synthesis gene clusters and the location of full-length IS elements to systematically compare the distribution of IS element groups in relation to individual SM synthesis gene clusters (or fragments). When comparing IS element net distance among the SM synthesis gene clusters, the majority of IS elements showed a non-normal distribution (Kolmogorov–Smirnov test *p* < 0.01). Notably, for nearly all IS element groups, the range in variation of net distance from a specific SM synthesis gene cluster exceeded 1 Mbp. Only for IS element group 3, a more narrow range in relation to the *pag* gene cluster was recorded, that is 0.12 Mbp. Therefore, any IS element group showed a clustering of its copies in the vicinity of a specific SM synthesis gene cluster. Nevertheless, a few copies of IS elements were found most closely located to a specific SM synthesis gene cluster (<11 kbp in distance), that is IS element group 2 (ISPlag1) for *mvd* genes, group 4 for *oci* and *apn* genes, group 3678 for *pag* genes, and group 1 and ISPlr1 for *mcy* genes.

**FIGURE 5:**
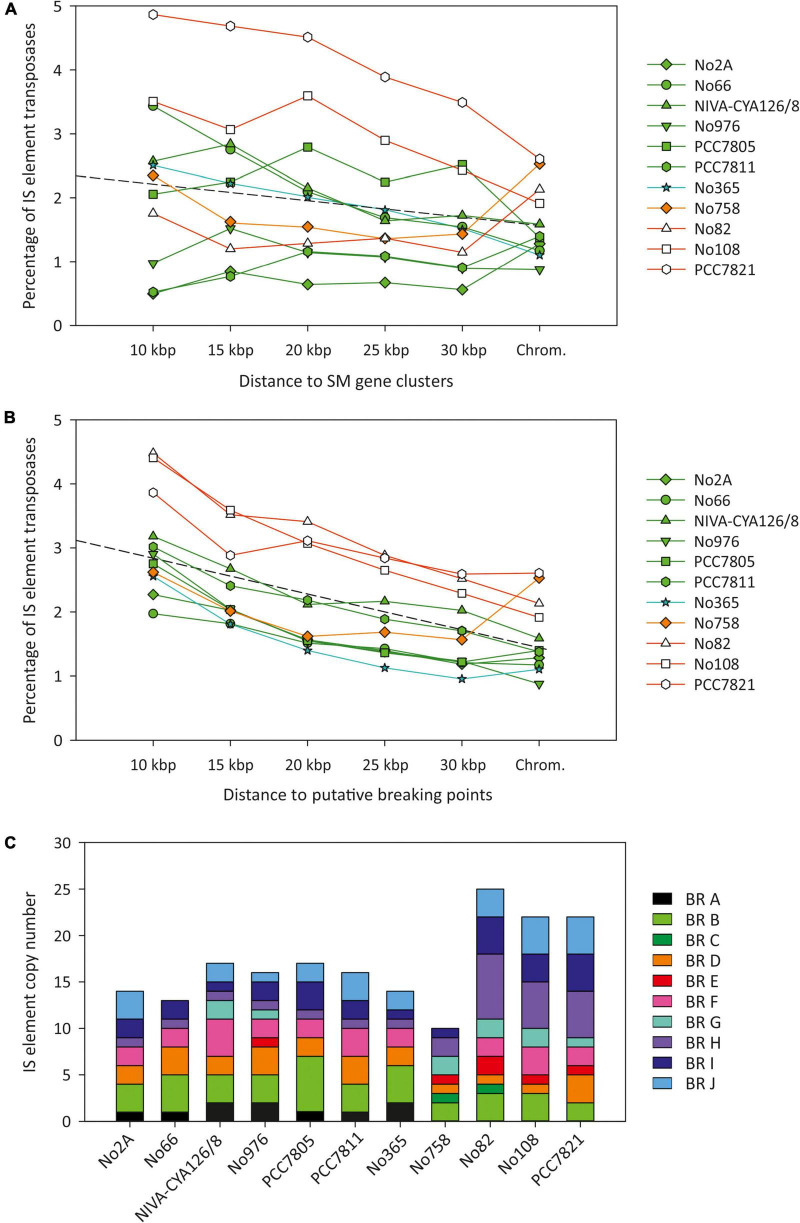
Relationship between IS element frequency (on a nucleotide basis) and the distance to **(A)** six SM synthesis gene clusters among eleven *Planktothrix agardhii*/*Planktothrix rubescens* chromosomes and **(B)** putative breaking regions for observed chromosomal rearrangements. **(C)** Frequency of IS element copy number within breaking regions (BR, putative breaking point ± 10 kbp).

We calculated the frequency of IS elements (including the remnants) in the adjacent regions (10–30 kbp) of the seven SM synthesis gene clusters (Figure 5A). In general, the strains did not show an increased IS element content in the vicinity of SM synthesis gene clusters when compared with the entire chromosome. Only PCC7821, No108, and No66 indicated an IS element frequency that increased in the vicinity of SM synthesis gene clusters.

However, the *pag* and *mic* gene clusters included single IS element copies. The *pag* gene cluster of seven strains included a transposase (Supplementary Additional File 3: Figure S5) as reported for *P. agardhii* NIES-596 and ISpagF previously (Donia and Schmidt, 2011). The two *mic* gene clusters included a specific transposase, i.e., for strain No66 an IS element of group 4 was found, and for strain PCC7821 ISPlr1 was located within the *mic* gene cluster. Only a few transposases were found adjacent to SM synthesis gene clusters, for example, ISPlag1 (group 2) was found close to the *apn* gene cluster (or *apn* remnants) in many strains of Lineages 1 and 1A. Therefore, relatively few IS elements were found in proximity to one of the seven SM synthesis gene clusters among *P. agardhii*/*P. rubescens* strains.

### Chromosomal Rearrangements Influencing (Co)Localization of Secondary Metabolites Synthesis Gene Clusters

**FIGURE 6:**
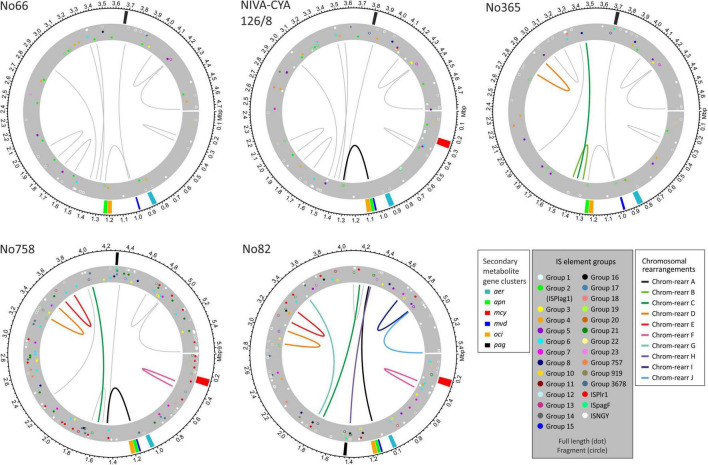
Location of secondary metabolites (SM) synthesis gene clusters and insertion sequence (IS) elements on chromosomes of *Planktothrix agardhii*/ *Planktothrix rubescens* strains, including No66 and NIVA-CYA126/8 (Lineage 1), No365 (Lineage 1A), No758 (Lineage 2A), and No82 (Lineage 2). Putative breaking regions of chromosomal rearrangements are indicated by drawn lines (circular plots for other strains have been included in Supplementary Additional File 3: Figure S7).

As mentioned previously, *P. agardhii* and *P. rubescens* strains showed a similar chromosomal structure. In total, ten major chromosomal rearrangements were observed (Figure 6 and Supplementary Additional File 3: Figure S7). We investigated the breaking regions of the ten chromosomal rearrangement events (approximately 30 kbp up and downstream of the putative breaking point) with regard to general gene composition, gene duplications, IS elements, and repetitive sequences (Supplementary Additional File 4: Tables S8–S16). In general, within putative breaking regions, we observed not only IS elements but also genes of the primary metabolism or uncharacterized genes.

**FIGURE 7:**
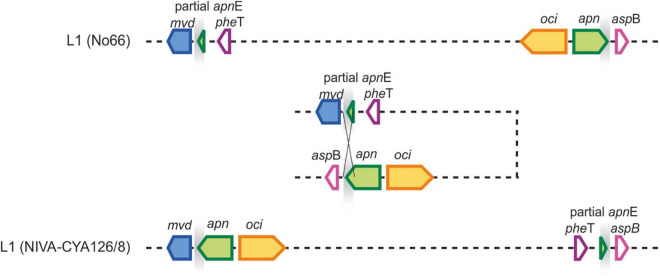
Chromosomal rearrangement A related to colocalization of *mvd* and *apn* gene clusters. Homologous gene loci are indicated by gray shading, which enabled the inversion of a 300 kbp fragment leading to a meta peptide gene cluster comprising *mvd*, *apn*, and *oci* genes.

The chromosomal rearrangements A and H influenced the chromosomal positions of some SM synthesis gene clusters and contained duplicate genes. As described previously, some strains of Lineage 1 had two copies of *apnE* (ABC transporter encoded by the *apn* gene cluster) possibly because of *apn* gene loss and regaining of the *apn* gene cluster (Entfellner et al., 2017). The presence of the entire *apn* gene cluster and the additional *apnE* was related to chromosomal inversion (approximately 270 kbp), moving the *apn* and *oci* synthesis gene clusters close to the *mvd* gene cluster and thus forming a meta gene cluster, namely, *mvd*–*apn*–*oci* (Figure 7), which is ubiquitously present among strains of Lineage 2. Alternatively, some strains in Lineage 1 (e.g., strain No66) lost the meta gene cluster formation by the same event. Notably, within Lineage 2, another chromosomal rearrangement H (approximately 2.3 Mbp) resulted in the movement of *pag* synthesis genes closer to the meta gene cluster (distance approximately 280 kbp).

In general, among the putative breaking regions, repetitive sequences were observed (Supplementary Additional File 4: Tables S9, S10, S14–S16): WD repeat-containing protein genes were found at the breaking regions of chromosomal rearrangements B, C, G, H, I, and J. In particular, for chromosomal rearrangement J, we found repetitive sequences at both and very close to the putative breaking points in all strains of Lineage 2. Repetitive sequences that are part of an unknown ORF encoding a tetratricopeptide protein were found at breaking regions of chromosomal rearrangements H and I.

Furthermore, we observed increased IS element frequency among the breaking regions. Notably, a correlation between IS element frequency (proportion of nucleotides) and the distance to the assumed breaking points of chromosomal rearrangements was observed (Figure 5B). The percentage of IS elements (transposases on nucleotide basis) among breaking regions was highest within 10 kbp up and downstream (1.9–4.5%) and lowest for the entire chromosome (0.9–2.6%), and it decreased with distance from breaking point. For all ten chromosomal rearrangements, at least one IS element was found, whereas most breaking regions showed several copies of IS elements (Figure 5C). Therefore, an increased proportion of IS elements occurred within breaking regions, which was related to chromosomal rearrangements predominantly within Lineage 2 (rearrangements C, E, and G–J) and to a less extent within Lineage 1 (rearrangements A, B, and D).

## Discussion

### Relationship of Genome Size and Insertion Sequence Element Proportion

Zhou et al. (2008) compared 1,356 IS elements from the IS finder database for 19 complete genome sequences of cyanobacteria and concluded that the genome size tends to increase with the number of recently active IS elements in a genome. In this study, not only full-length copies but also fragments were found to correlate, which might be due to the presence of repetitive sequences. Similarly, Larsson et al. (2011) reported a highly significant correlation between genome size and the number of duplicated genes. When comparing 58 cyanobacterial genomes, the authors concluded that numerous paralogs (resulting from gene duplication) assigned to COG L (replication) were mostly linked to transposases. In general, transposases (IS elements) could be used to create/maintain genomic plasticity, thereby potentially allowing rapid adaptation to environmental changes (Frangeul et al., 2008; Kusumoto et al., 2011; Humbert et al., 2013; Nzabarushimana and Tang, 2018). Details on IS-based molecular adaptation mechanisms are less clear, but partial IS elements rather facilitate homologous recombination processes because of sequence similarity than actively inducing it by cut and paste mechanism.

By contrast, in this study, the chromosome size slightly increased with the frequency of IS elements, and the content of other paralogs was smaller than that of IS elements. Thus, likely orthologous genes inserted by HGT led to genome increase during evolution of Lineage 2. A large share of these orthologous genes was composed of uncharacterized hypothetical proteins (Table 2). However, Tooming-Klunderud et al. (2013) demonstrated earlier that the strains assigned to *P. rubescens* gained a gene cluster encoding phycoerythrin synthesis through HGT, which is considered as a selective advantage in deep stratified lakes (Kurmayer et al., 2015). Understanding the physiological function of the acquired total genetic basis ranging from 0.8 to 0.995 Mbp is considered important to understand the ecophysiological adaptation of Lineage 2 on a genomic level.

According to the botanical definition (Anagnostidis and Komárek, 1988) the green vs red pigmentation is the major criterion to differ between *P. agardhii* and *P. rubescens* (Suda et al., 2002). The two major phylogenetic lineages identified through multi-locus sequence analysis (MLSA) previously were confirmed via phylogenomic analysis in this study (Figure 1 and Supplementary Additional File 3: Figure S2). However, as found earlier both lineages comprise green and red pigmented strains not reflecting the current species definition according to Suda et al. (2002). HGT of phycoerythrin synthesis only is unlikely to be the sole factor for evolutionary diversification. On the other hand the observed high genetic dissimilarity between phylogenetic Lineages 1 and 2 points to a more stable phylogenetic barrier which would imply genetic differentiation even if genotypes from both lineages occur in the same habitat (Jain et al., 2018). In the future for Lineage 2 the functional consequences of the acquired additional genetic information need to be explored concerning its role in the observed phylogenomic differentiation.

### Secondary Metabolites Synthesis Gene Clusters Influenced by Mobile Elements

In general, the observed patchy SM synthesis gene cluster distribution among genera/strains has been considered the result of mobile elements, that is plasmids and transposases (i.e., Bolch et al., 1997; Nakasugi et al., 2007). Based on this study, most SM synthesis gene clusters have been stably integrated into the chromosome, which show a distribution congruent to phylogeny. Moreover, within Lineage 2, all four strains contained at least six SM synthesis gene clusters (*aer*, *mvd*, *apn*, *oci*, *pag*, and *mcy*) probably because an ancestor genotype already carried all of these clusters. By contrast, the number of SM synthesis gene clusters among Lineage 1 was reduced because of gene loss processes possibly influenced by IS elements (loss of *mcy* and *apn* genes related to insertion of ISPlag1). Only the *mic* gene cluster has been found on a plasmid, which may be the first example of a plasmid encoding a functional SM synthesis gene cluster for bloom-forming cyanobacteria (strains No66, PCC7821). However, for the genus *Planktothrix*, the genomic mobility of the six most abundant SM synthesis gene clusters is considered low.

Similar to plasmids, various IS elements located within or in the vicinity of SM synthesis gene clusters have been suggested to facilitate HGT (e.g., Tillett et al., 2000; Christiansen et al., 2003). Correspondingly, in this study, for all SM synthesis gene clusters, a few IS elements were found closely located (<10 kbp), for example, strain No66 carried ISPlag1 and IS element group 4 flanking the *apn* gene cluster in a distance of 4 kbp. ISPlag1 also was found in the vicinity of *apn* gene clusters in strains No2A and No365. Considering that *apn* genes might have been re-introduced through HGT recently (Entfellner et al., 2017), the IS elements, namely, ISPlag1 and IS group 4, might have been involved. Two IS elements were part of the *mic* gene cluster, that is IS group 4 (No66) and ISPlr1 (PCC7821). Considering that the percentage of IS elements on plasmids ranged from 0 to 16.4%, the IS element share on plasmids is generally more flexible when compared with the chromosome. If the incoming DNA contains parts similar to the recipient plasmid/chromosome such as IS elements (Herrero and Flores, 2008), then IS elements present in plasmids can serve as the recognition site for homologous recombination.

Based on another hypothesis, active IS elements might cluster in host genomes (Zhou et al., 2008). Such clustering may affect the mutation rate in certain regions of the host genome, including functional operons such as SM synthesis gene clusters. Indeed, in this study, increased IS element frequency was observed in certain chromosomal regions related to chromosomal rearrangements (Figure 5B). It is not known whether this increased frequency in certain regions is because of (unknown) factors directing IS element insertion or actually occurs accidentally. Nevertheless, we argue that the physical vicinity of IS elements to SM synthesis gene clusters is not of decisive importance for modification, rather it is the activity of certain IS elements (ISPlr1 and ISPlag1). For example, IS element group 1 is found closely located to the *mcy* gene cluster (except No758), but it shows no influence. Therefore, the (active) IS elements affect certain SM synthesis genes (*mcy*) from the distance, for example, directed by the presence of short repetitive (RR 1–7, 41–46 bp) sequences (Chen et al., 2016). Such RR sequences resemble the repetitive extragenic palindromic DNA sequences (REPs) described from many bacteria (Tobes and Ramos, 2005). REP sequences can form stem–loop hybridization during DNA replication, and they might direct transposases such as ISPlr1 (Tobes and Pareja, 2006). Notably, in contrast to *mcy* genes containing REPs (RR2,3,5,6,7, 43–46 bp), other SM synthesis gene clusters located in the chromosome of *Planktothrix* did not contain these re-occurring sequence motifs, indicating a potential protection from ISPlr1 disruption (Rainer Kurmayer, unpublished data). For the majority of ISPlr1 copies, a REP sequence is found in the flanking region of the IS element (< 10 kbp distance, i.e. for 35 copies of the 40 copies in strains PCC7821, No82, No108, and No758).

### Evolution of Secondary Metabolites Synthesis Gene Cluster Localization in Chromosome

The colocalization of SM synthesis gene clusters (*mvd*–*apn*–*oci*) resulting in the meta peptide synthesis gene cluster has been reported from *Planktothrix* and has been considered as a genomic island (Rounge et al., 2009; Pancrace et al., 2017). Genomic islands, including several SM synthesis gene clusters, have been described from other bacteria, that is actinobacteria of the genus *Salinispora* (Penn et al., 2009; Ziemert et al., 2014). In general, such genomic islands have been considered as a result of transmittance, that is pathogenicity islands containing biosynthesis gene clusters for yersiniabactin (Fischbach et al., 2008), and a high frequency of mobile (IS) elements has been reported (Penn et al., 2009). In this study, the usual criteria for the differentiation of genomic islands were not met, that is neither a high share of mobile elements, nor GC percentage deviation, or codon usage bias could be found (data not shown). However, based on this criteria, all of the six chromosomally encoded SM synthesis gene clusters occurred already in the ancestor of *P. agardhii*/*P. rubescens*, and they were partly lost during the evolution of Lineage 1 (e.g., MC or anabaenopeptin). Recently, Pancrace et al. (2017) described the draft genome of a benthic *Planktothrix* strain PCC11201, which contained SM synthesis gene clusters for MC, aeruginosin, cyanopeptolin, microviridin, and prenylagaramide. In this study, *P. agardhii* strains, namely, PCC7805 and No976, represented genotypes that lost the *apn* gene cluster previously but kept *apnE* as a remnant in the flanking region of the *mvd* gene cluster (Entfellner et al., 2017). Similarly, strains such as No2A, No66, No976, No365, PCC7805, and PCC7811 still contained remnants of *mcyT* indicative of the former *mcy* gene cluster (Kurmayer et al., 2016). Notably, other genotypes such as NIVA-CYA126/8 maintained the six SM biosynthesis gene clusters, which can be considered representative of the ancestor of Lineages 1 and 2.

Aside from SM synthesis gene clusters, the presence or absence of chromosomal rearrangement processes led to a localization in the chromosome that was congruent with phylogeny (Figure 4), that is three strains of Lineage 2 showed a colocalization of six SM synthesis gene clusters within 1 Mbp. By contrast, strain NIVA-CYA126/8 and strain No758 still carried the *pag* gene cluster more distant from other SM synthesis gene clusters. At present, we can only speculate, but in general, the clustering of genes has been related to coordinate regulation, that is to activate a specific pathway on demand (Fischbach and Walsh, 2006). In addition, the colocalization of several SM synthesis pathways might aid in regulation, for example, in *P. agardhii*, NRPS and RiPP peptides are coproduced (Welker et al., 2004). Rohrlack and Utkilen (2007) reported that anabaenopeptin versus microviridin are produced constitutively despite marked changes in culture conditions. In general, those peptides are produced in high intracellular amounts approaching 1% of dry weight (Jüttner and Lüthi, 2008; Kosol et al., 2009). Such high concentrations may have intracellular effects, such as the covalent binding of MC variants to the free cysteine groups of abundant proteins (phycobilins) through the methyl-dehydroalanine group in pos. 7 of the MC molecule (Zilliges et al., 2011). Thus, the colocalization of NRPS and RiPP gene clusters might facilitate the regulation of individual peptide synthesis pathways, resulting in a more balanced intracellular peptide concentration.

## Conclusion

The microevolution of the bloom-forming cyanobacteria *Planktothrix* spp. is related to a considerable variation in chromosome size. This variation in chromosome size, spanning almost 2,000 kbp, has been caused by gene duplication of IS elements or other genes to a minor extent only, while gene deletion and HGT events contributed more than eighty percent. A few functional adaptations and poorly characterized proteins including transposases were acquired by HGT. Six of seven peptide synthesis gene clusters occurred already in the ancestor of *P. agardhii/P. rubescens*, and became partly lost during the evolution of Lineage 1. Overall, IS elements have been involved in SM gene cluster loss processes, however, did not occur more frequently in the vicinity of SM synthesis gene clusters. By contrast, IS elements have been observed more frequent within breaking regions causing chromosomal rearrangements, thereby influencing the colocalization of SM synthesis gene clusters on the chromosome.

## Data Availability Statement

The genomic data presented in this study are deposited in the EMBL-EBI repository, under STUDY_ID PRJEB40445 (ERP124090). Genbank accession numbers: NIVA-CYA126/8, GCA_904830765, LR882934-LR882937; No2A, GCA_904830775, LR882938-LR882940; No66, GCA_904830855, LR882963-LR882966; No82, GCA_904848565, LR890048-LR890051; No108, GCA_904830785, LR882941-LR882941; No365, GCA_904830845, LR882944-LR882948; No713, GCA_904830925, LR882967-LR882968; No758, GCA_904830945, LR882949-LR882949; No976, GCA_904830935, LR882952-LR882957; PCC7805, GCA_904830915, LR882950-LR882951; PCC7811, GCA_904830885, LR882969-LR882969; PCC7821, GCA_904830895; LR882958-LR882962; PCC9214, GGCA_904830955, LR882970–LR882972.

## Author Contributions

EE, RL, and RK performed the data analysis and drafted the manuscript. YJ and LD confirmed the plasmids by PCR analysis. JR and LD aligned the contigs from raw sequences. JB and RK assisted in exploitation of sequence information such as annotation, COG analysis and paralog identification and sequence submission to GenBank. RK supervised the work and reviewed the manuscript. All authors contributed to the article and approved the submitted version.

## Conflict of Interest

The authors declare that the research was conducted in the absence of any commercial or financial relationships that could be construed as a potential conflict of interest.

## Publisher’s Note

All claims expressed in this article are solely those of the authors and do not necessarily represent those of their affiliated organizations, or those of the publisher, the editors and the reviewers. Any product that may be evaluated in this article, or claim that may be made by its manufacturer, is not guaranteed or endorsed by the publisher.

## Footnotes

1
https://github.com/PacificBiosciences/ccs

2
http://www.microbesonline.org/fasttree
