# Supplementary material for: Toxic/Bioactive Peptide Synthesis Genes Rearranged by Insertion Sequence Elements Among the Bloom-Forming Cyanobacteria Planktothrix
Source: Front Microbiol. 2022 Jul 28;13:901762. doi: 10.3389/fmicb.2022.901762 (PMC9366434; doi:10.3389/fmicb.2022.901762)
Supplement: Supplementary file 2 [file Data_Sheet_2.docx]

**Toxic/Bioactive Peptide Synthesis Genes Rearranged by Insertion Sequence Elements Among the Bloom-forming Cyanobacteria *Planktothrix***

Front. Microbiol.

Sec. Evolutionary and Genomic Microbiology

doi: 10.3389/fmicb.2022.901762

**Elisabeth Entfellner^1†^, Ruibao Li^1,2,3†^, Yiming Jiang^2^, Jinlong Ru^2^, Jochen Blom^4^, Li Deng^2^, Rainer Kurmayer^1*^**

^1^ Research Department for Limnology, University of Innsbruck, Mondsee, Austria

^2^ Institute of Virology, Helmholtz Zentrum München, München, Germany

^3^ Department of Ecology and Institute of Hydrobiology, Jinan University, Guangzhou, China

^4^ Bioinformatics and Systems Biology, Justus-Liebig-University, Giessen, Germany

^†^ These authors have contributed equally to this work and share first authorship

*** Correspondence:**Rainer Kurmayer
[rainer.kurmayer@uibk.ac.at](mailto:rainer.kurmayer@uibk.ac.at)

**Additional File 2:** Circular plots for plasmids which were physically confirmed by overlapping long-distance PCR and corresponding PCR products visualized by standard gel electrophoresis.

| No2A_plasmid 1  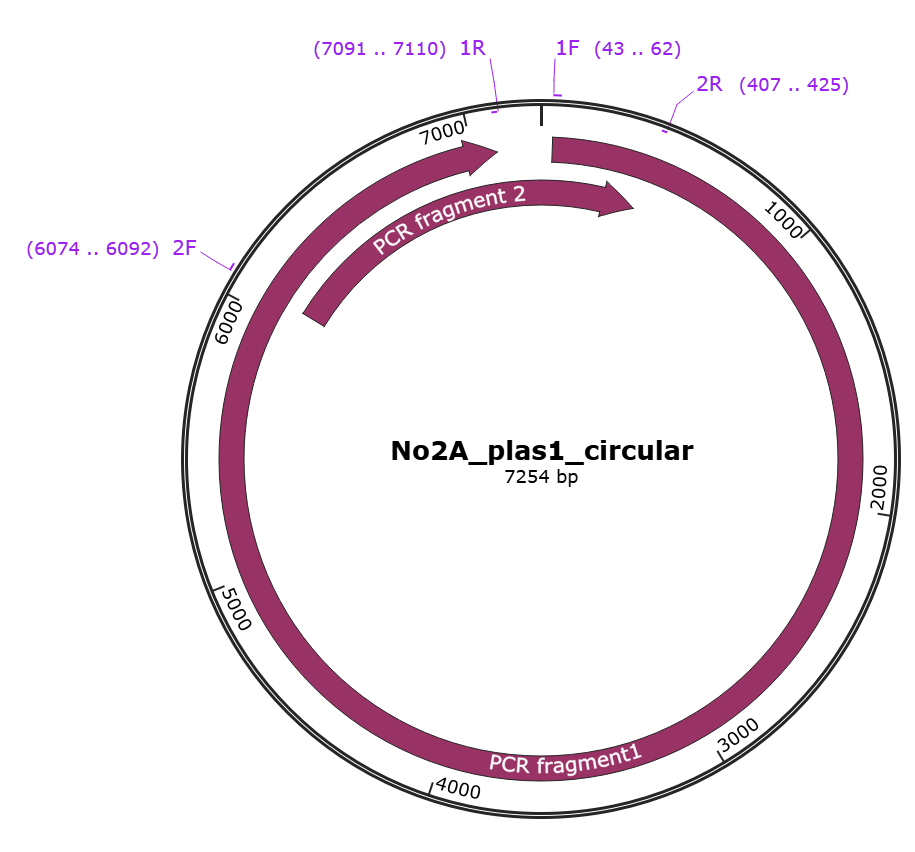 | PCR image (Marker: λ PstI)  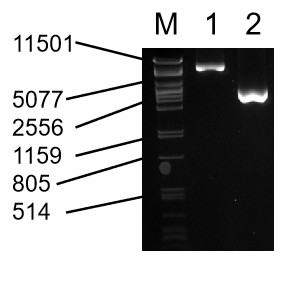 |
| --- | --- |
| No2A_plasmid 2  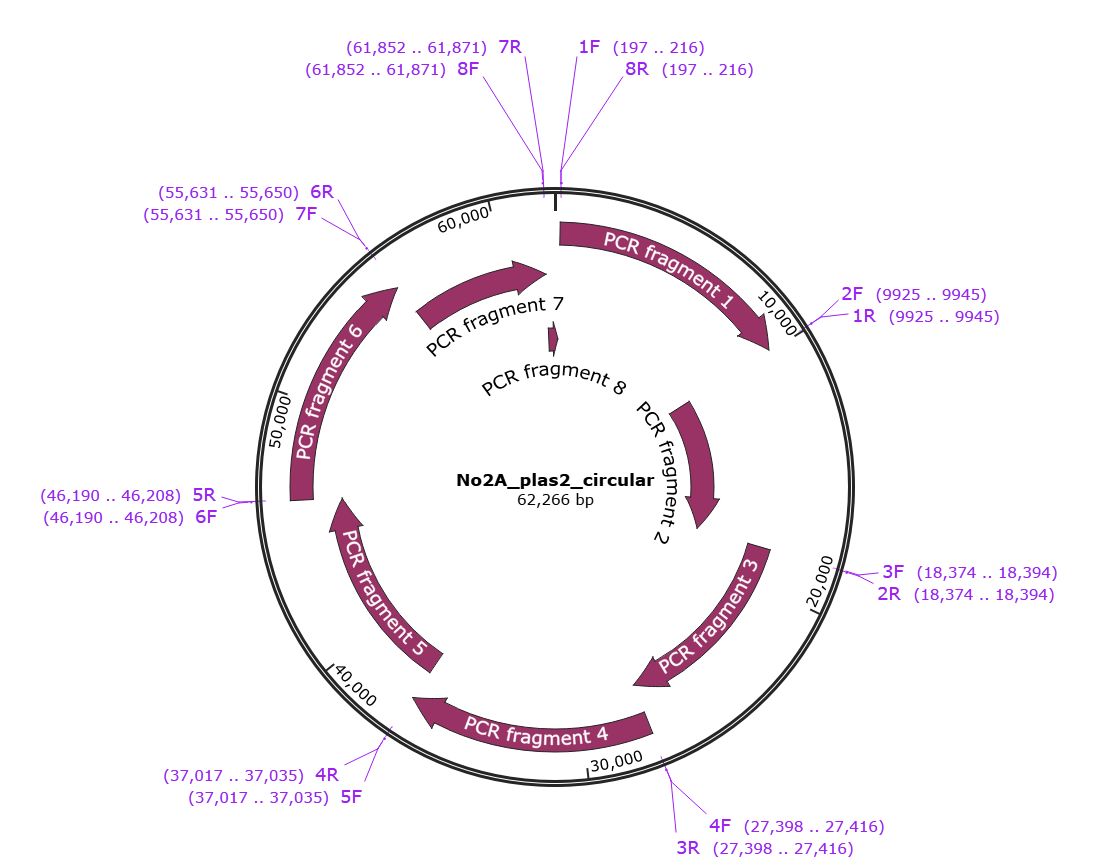 | PCR image (Marker: λ PstI)  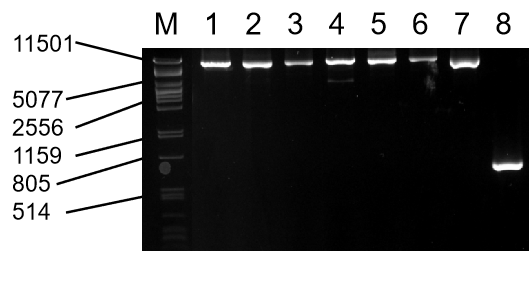 |
| No66_plasmid 2  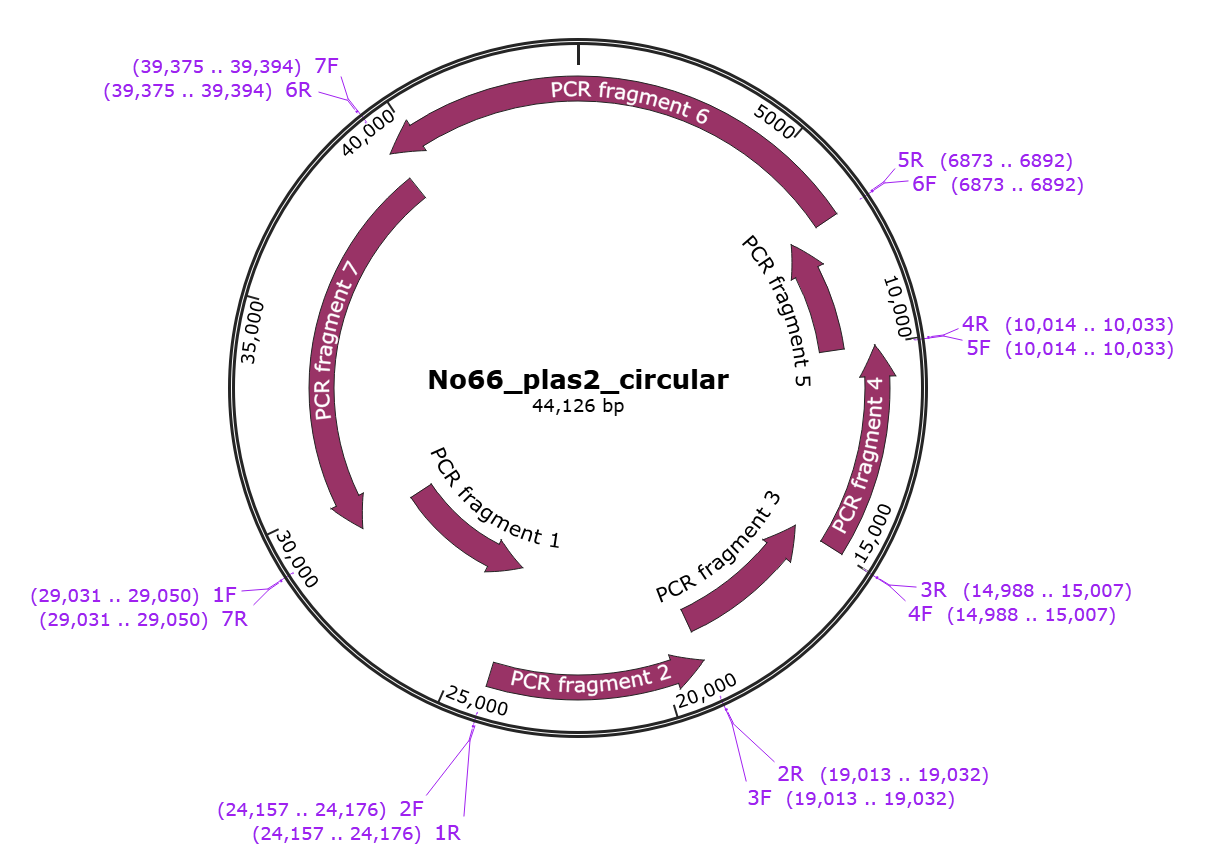 | PCR image (Marker: Generuler 1kb):  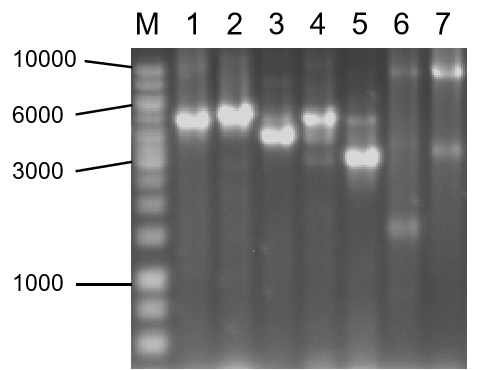 |
| No66_plasmid 3  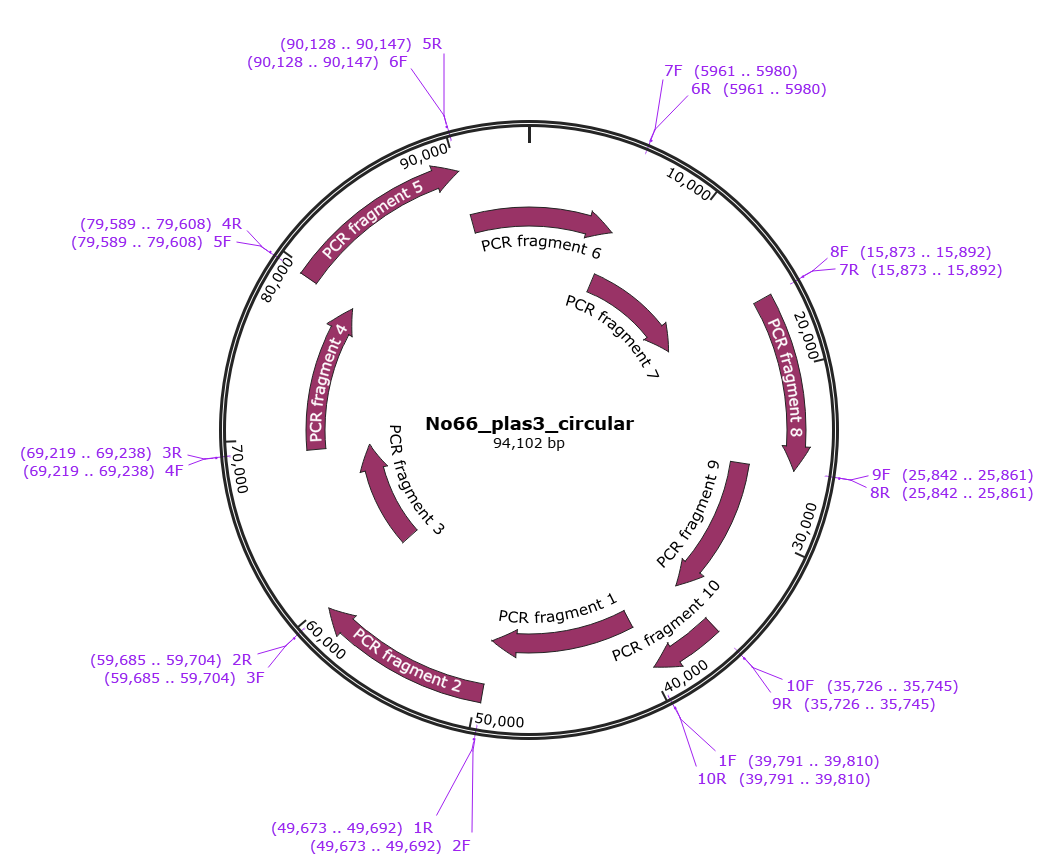 | PCR image (Marker: Generuler 1kb):  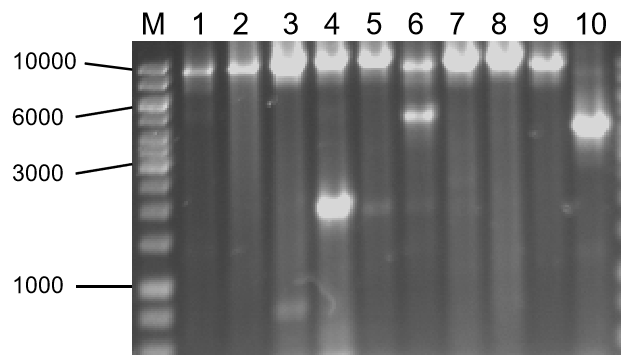 |
| No976_plasmid 1  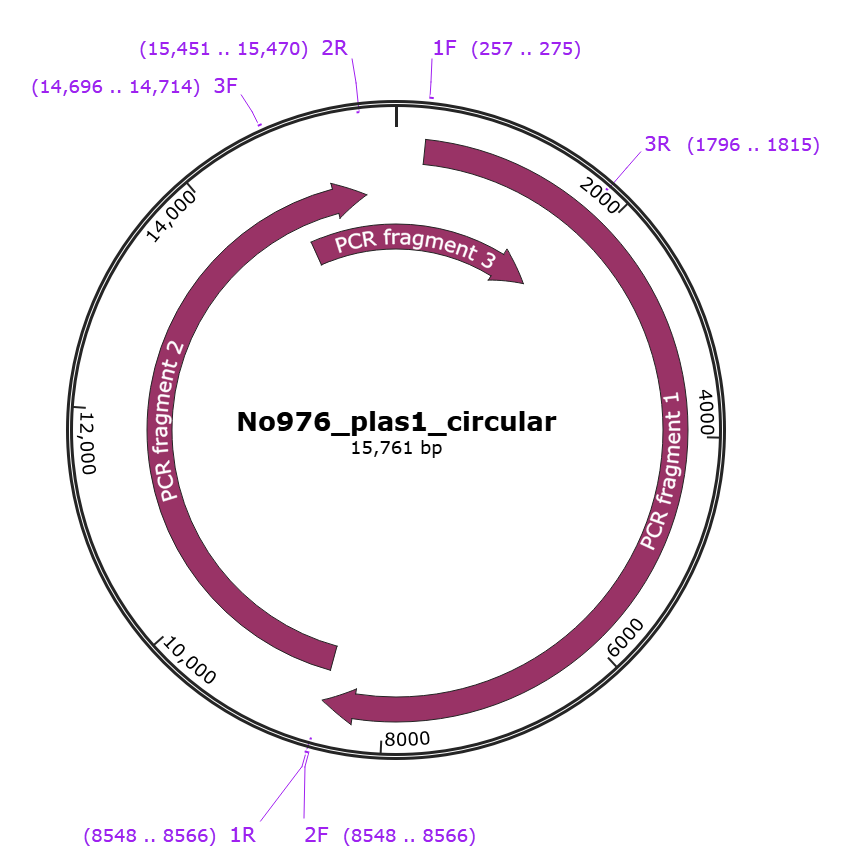 | PCR image (Marker: λ PstI)  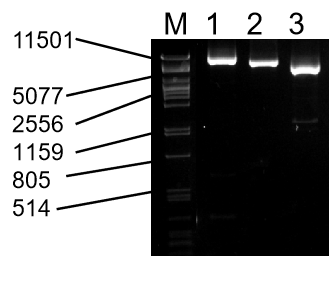 |
| No976_plasmid 2  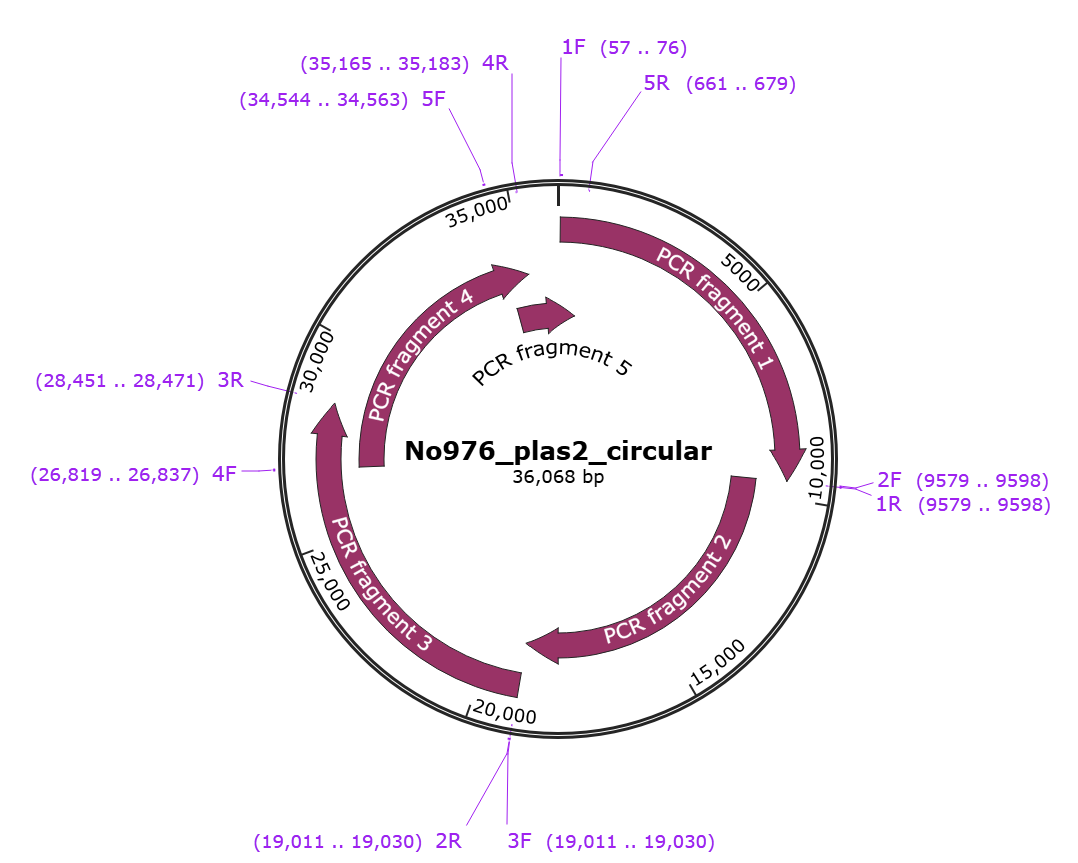 | PCR image (Marker: λ PstI)  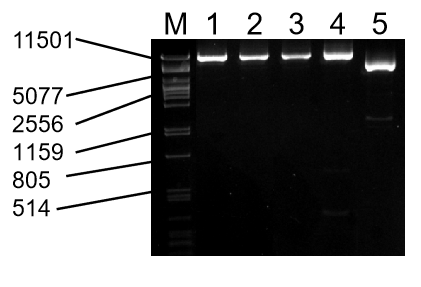 |
| No976_plasmid 3  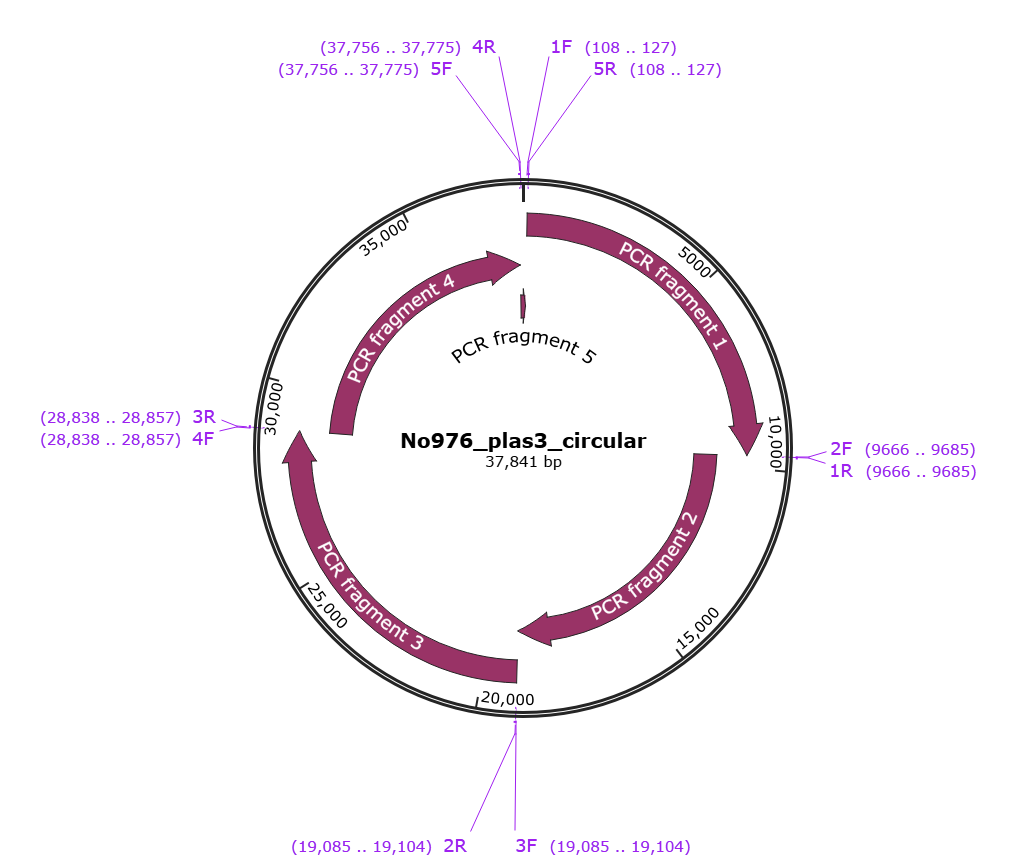 | PCR image (Marker: λ PstI)  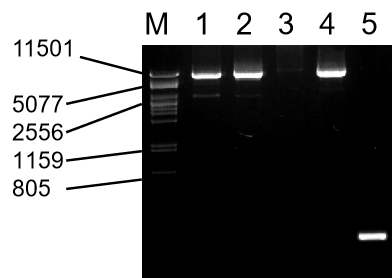 |
| No976_plasmid 4  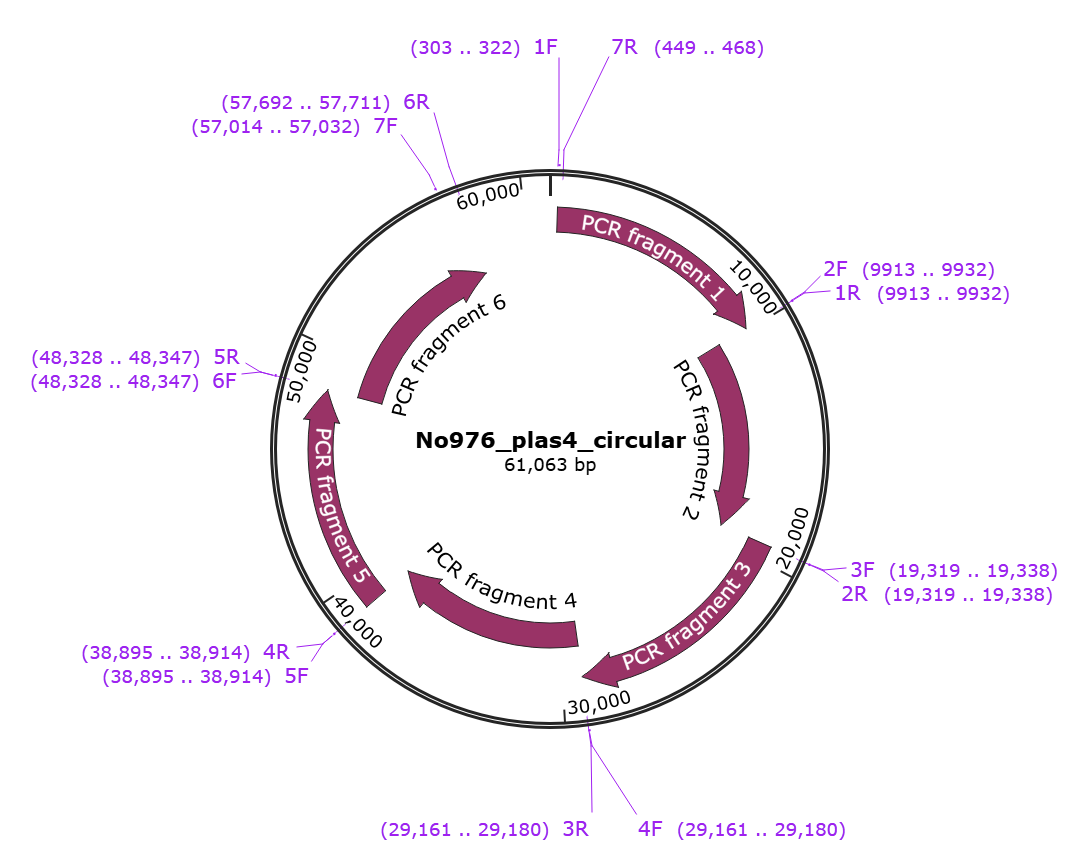 | PCR image (Marker: λ PstI)  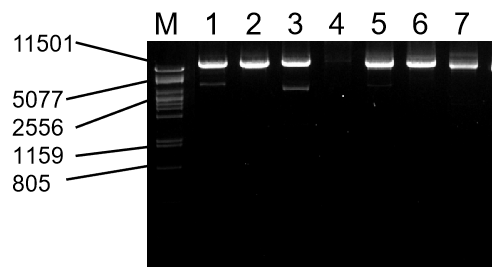 |
| No976_plasmid 5  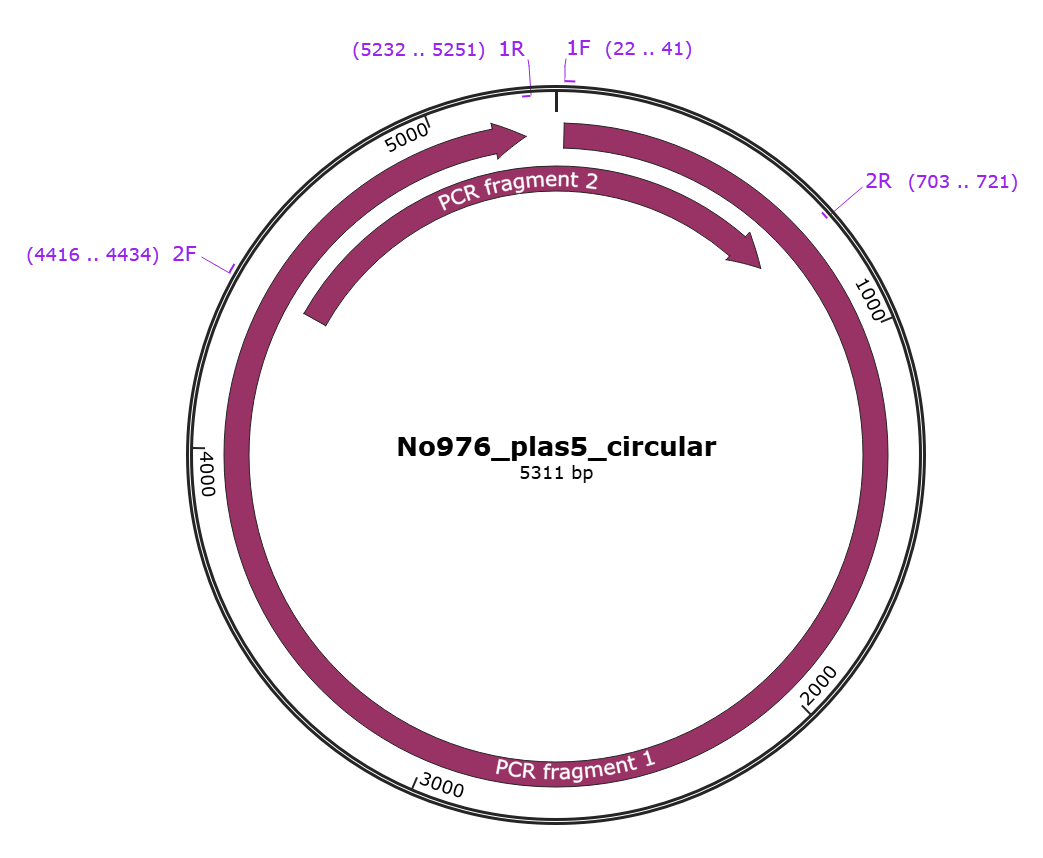 | PCR image (Marker: λ PstI)  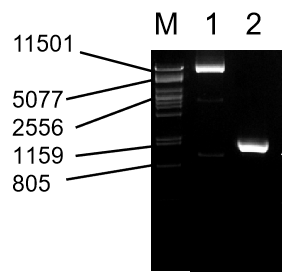 |
| PCC7805_plasmid  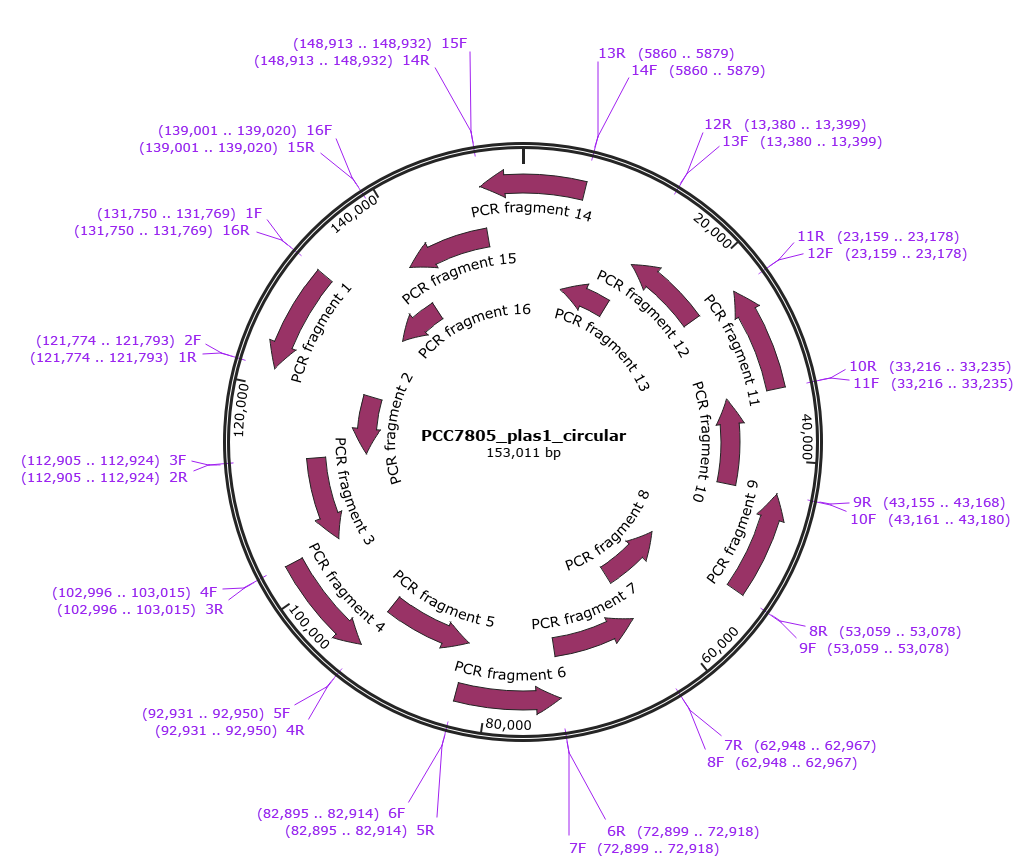 | PCR image (Marker: Generuler 1kb):  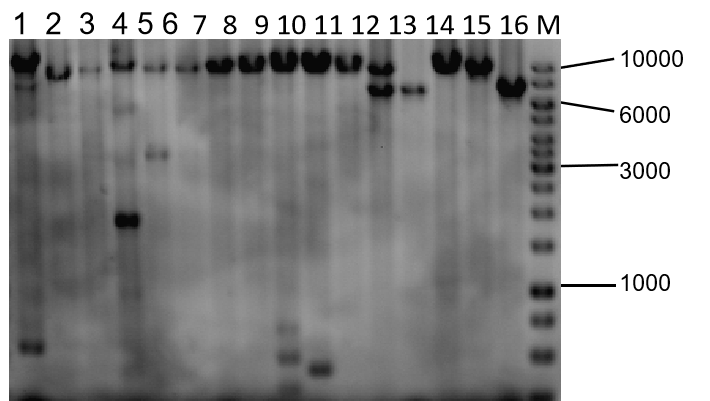 |
| No365_plasmid 1  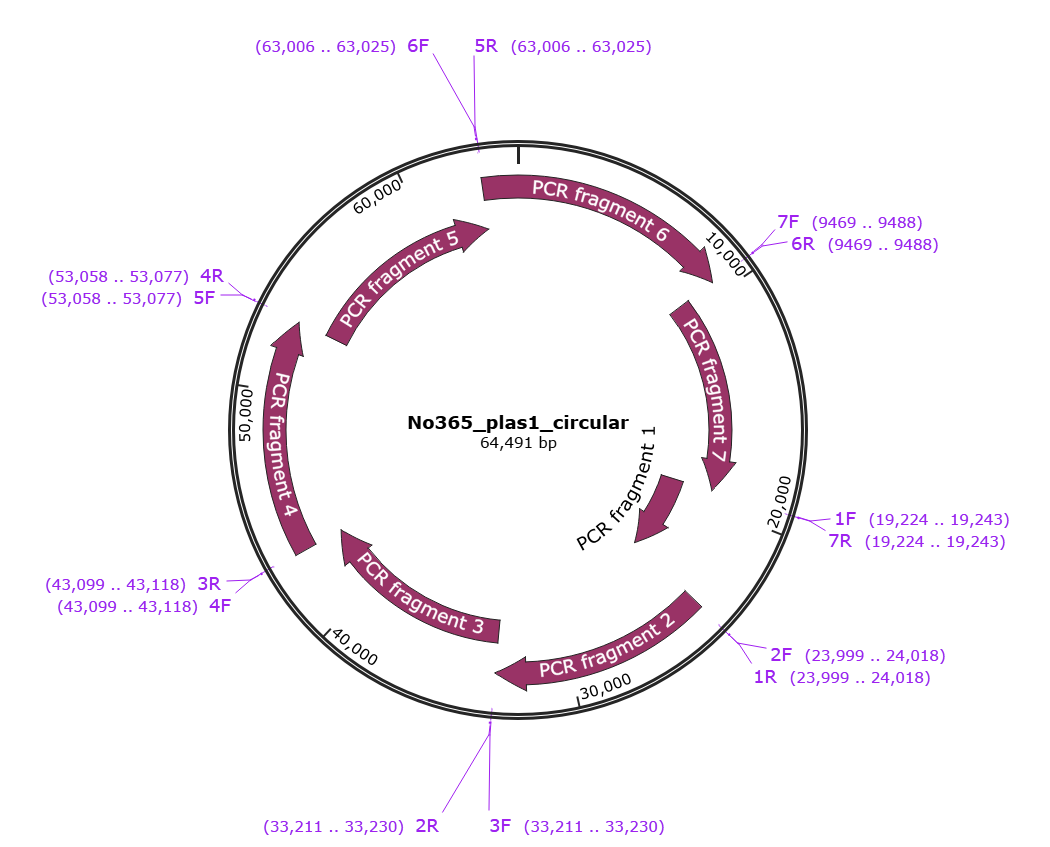 | PCR image (Marker: Generuler 1kb):  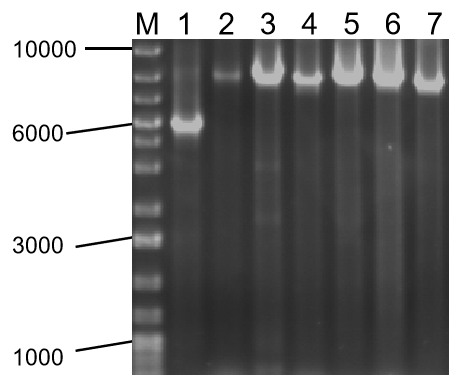 |
| No365_plasmid 2  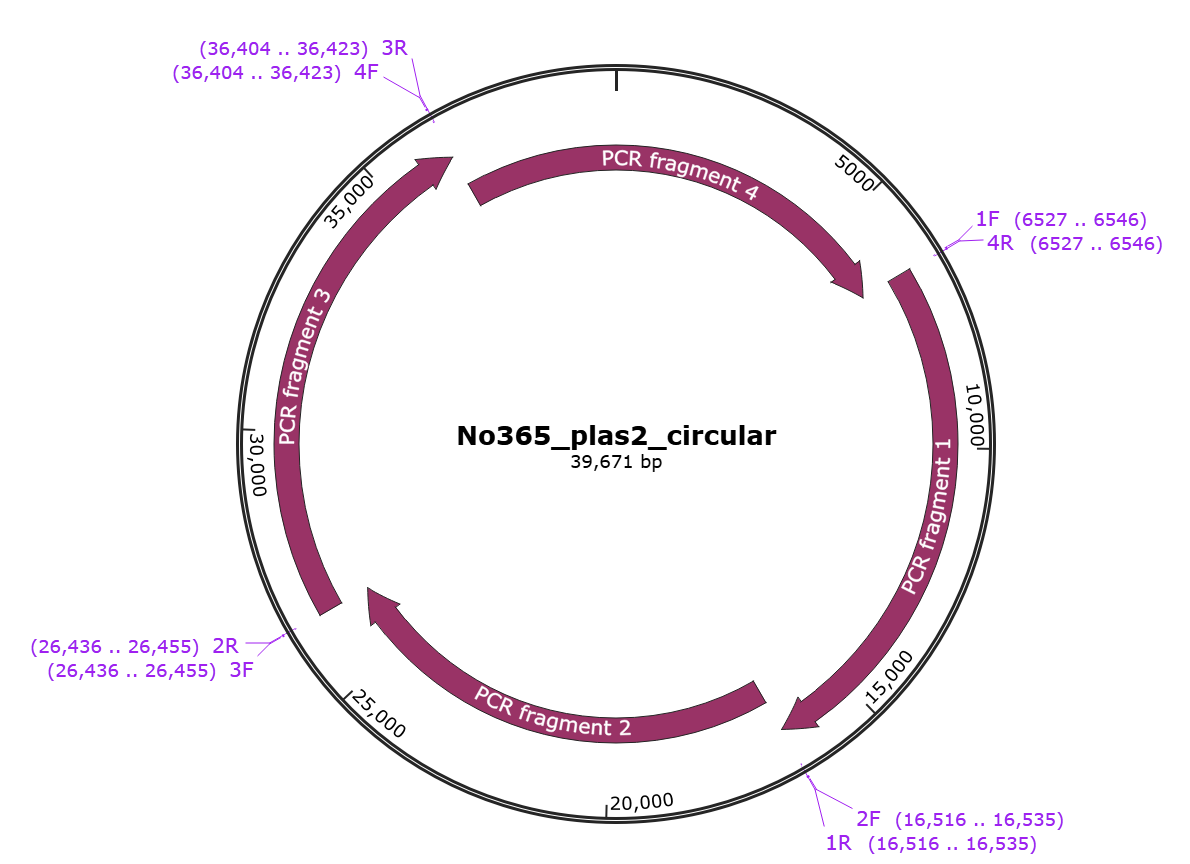 | PCR image (Marker: Generuler 1kb):  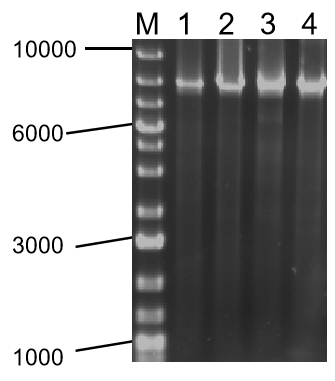 |
| No365_plasmid 3  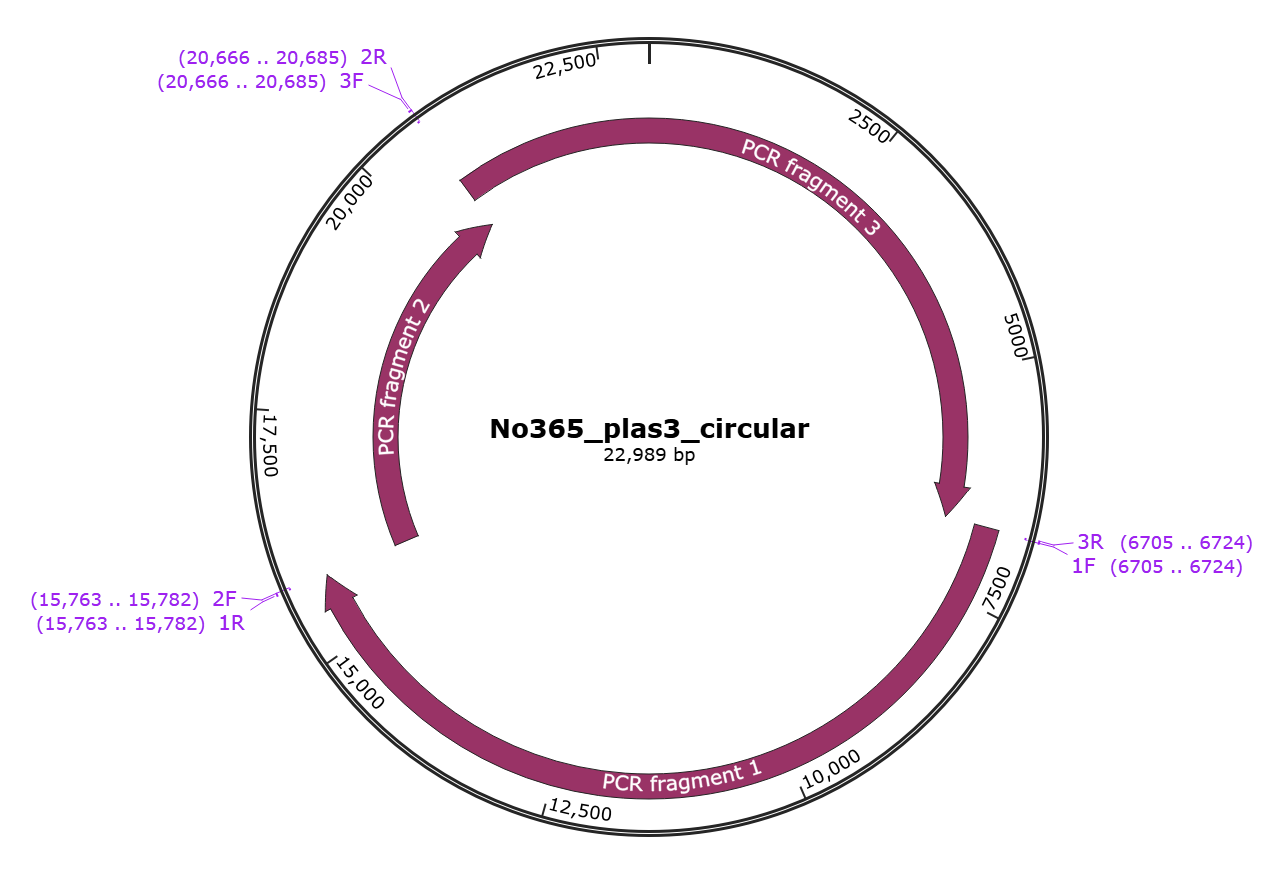 | PCR image (Marker: Generuler 1kb):  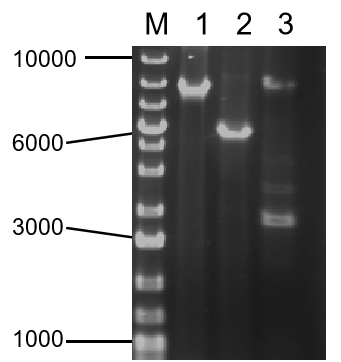 |
| No82_plasmid 2    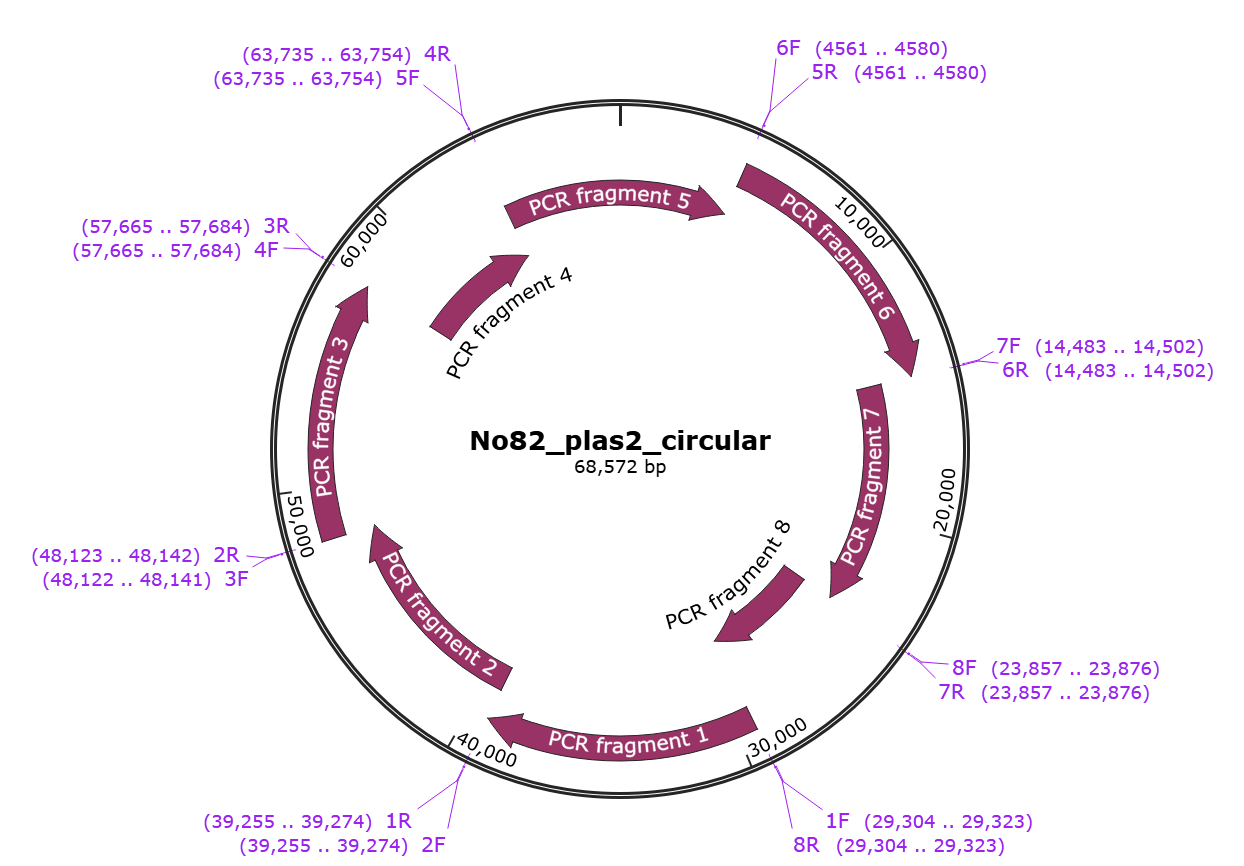 | PCR image (Marker: Generuler 1kb plus):  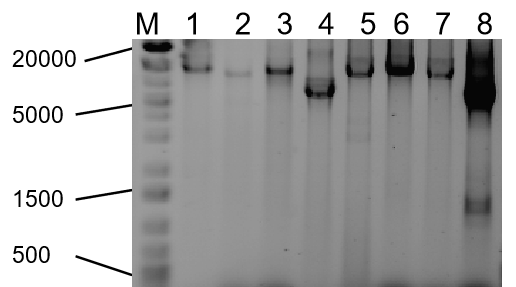 |
| No82_plasmid 3  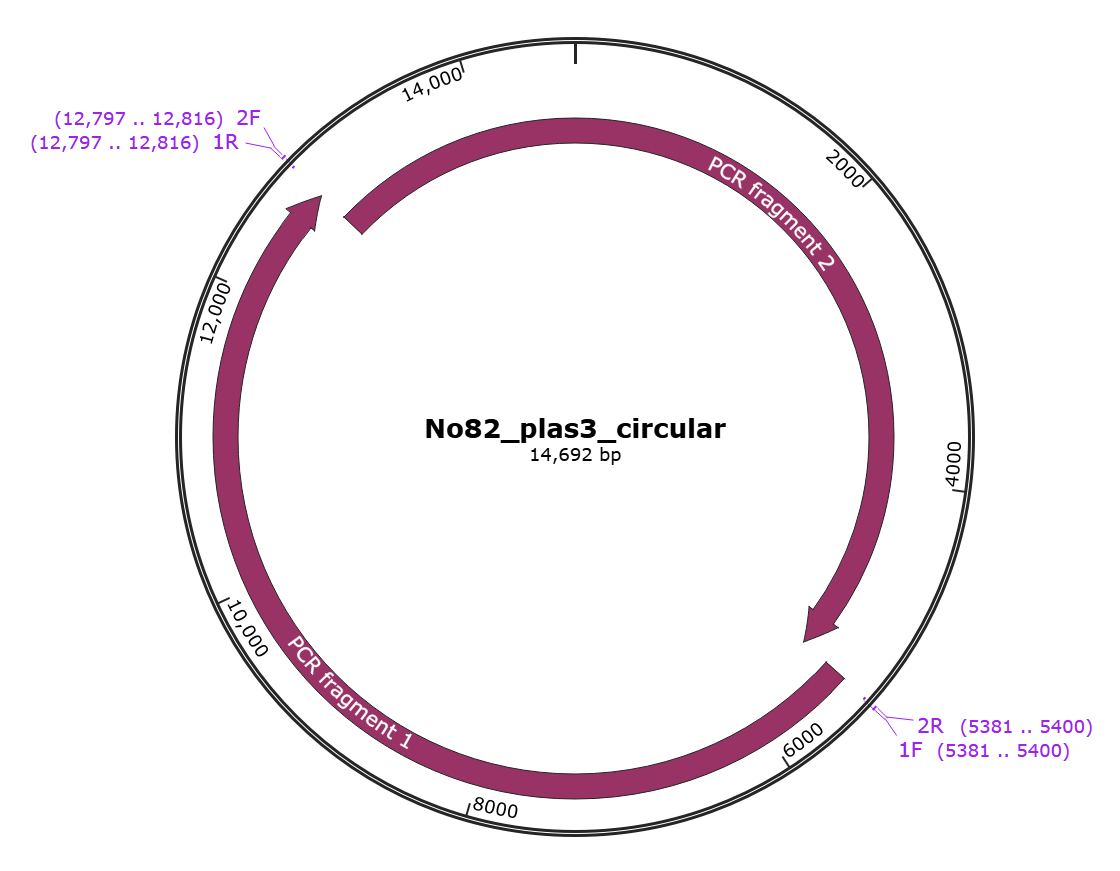 | PCR image (Marker: Generuler 1kb plus):  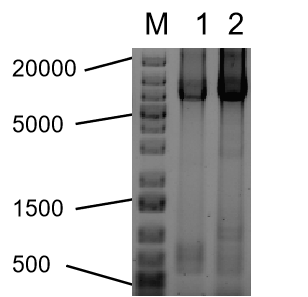 |
| PCC7821_plasmid 1  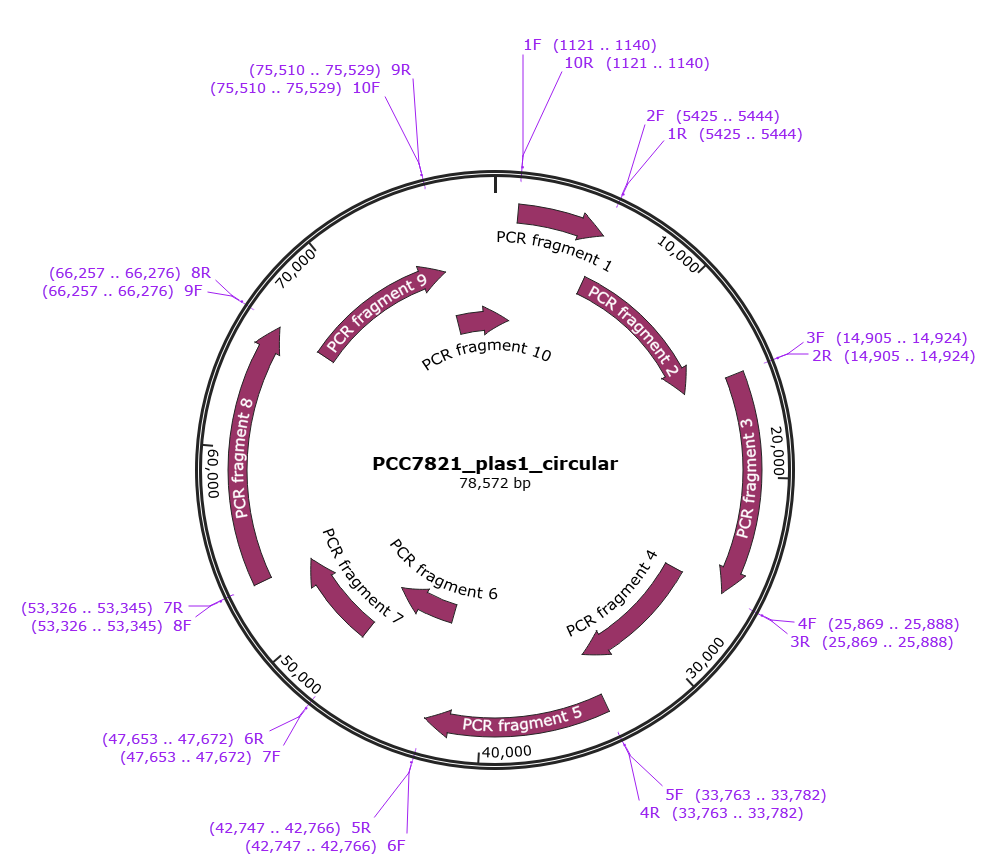 | PCR image (Marker: Generuler 1kb):  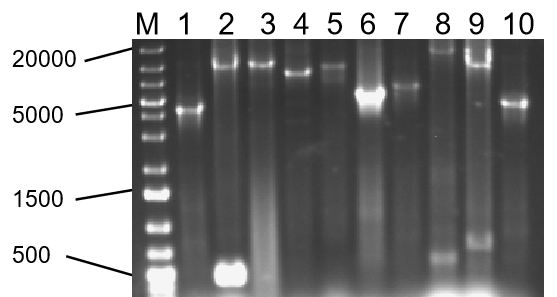 |
| PCC7821_plasmid 2  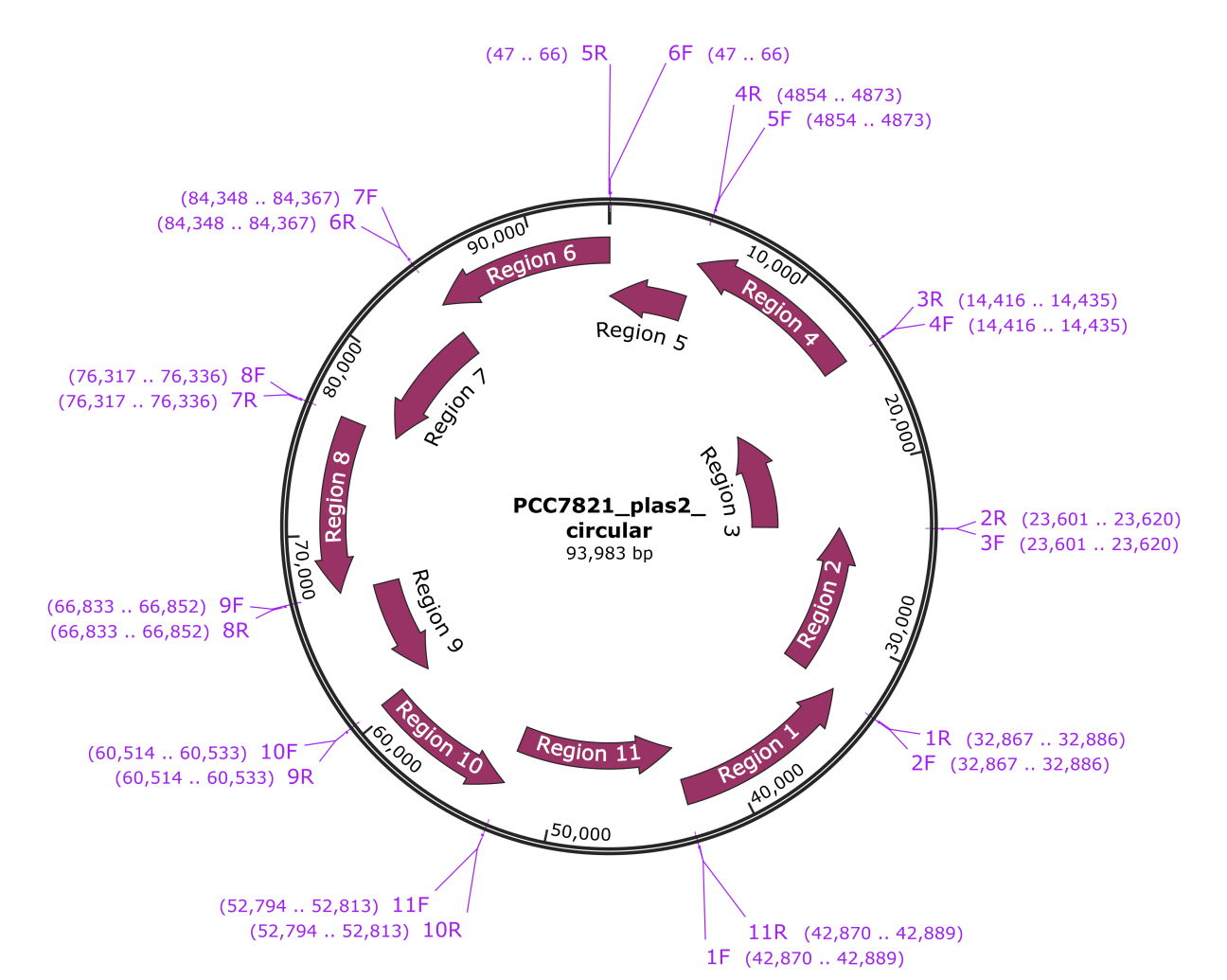 | PCR image (Marker: Generuler 1kb plus):  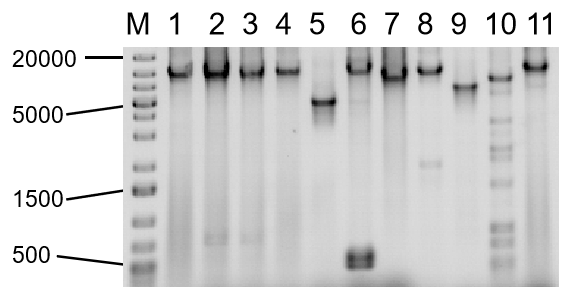 |
| PCC7821_plasmid 3  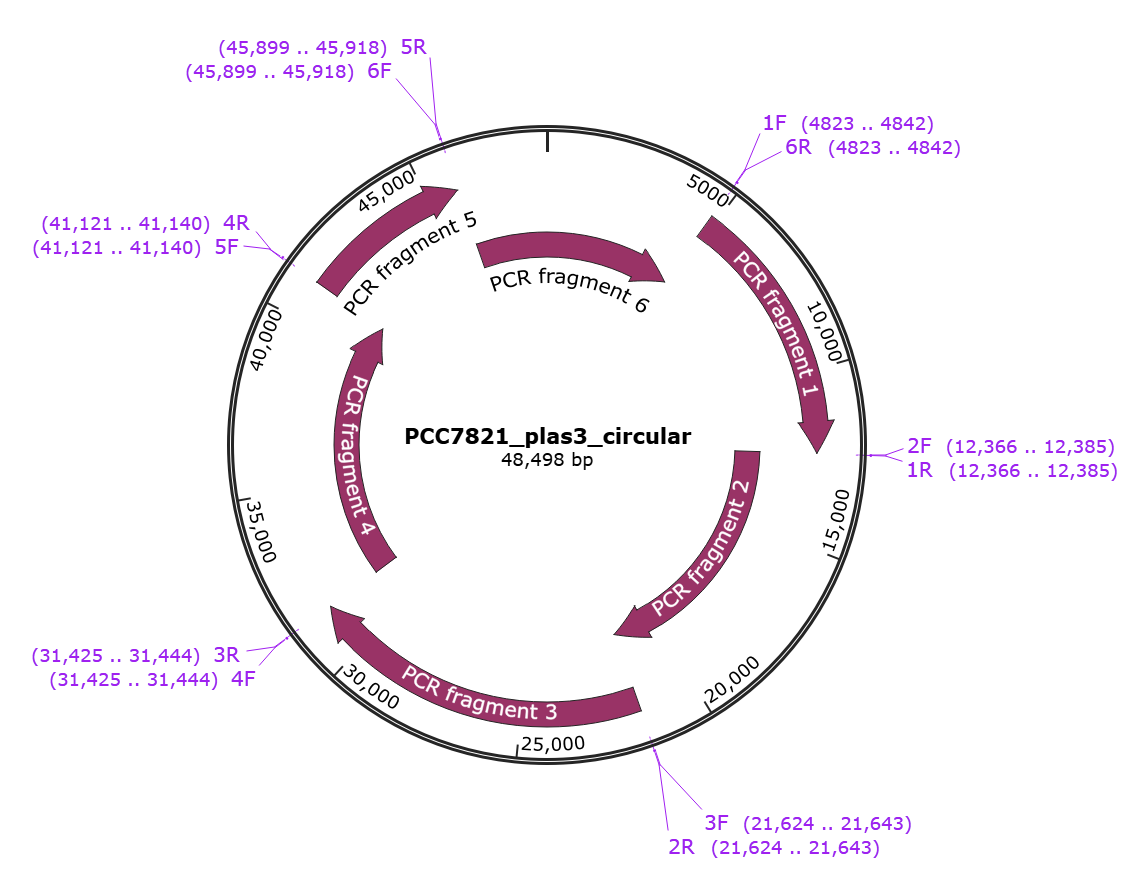 | PCR image (Marker: Generuler 1kb plus)  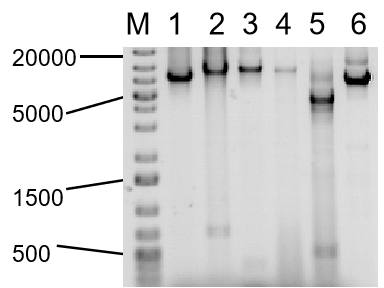 |
| PCC7821_plasmid 4  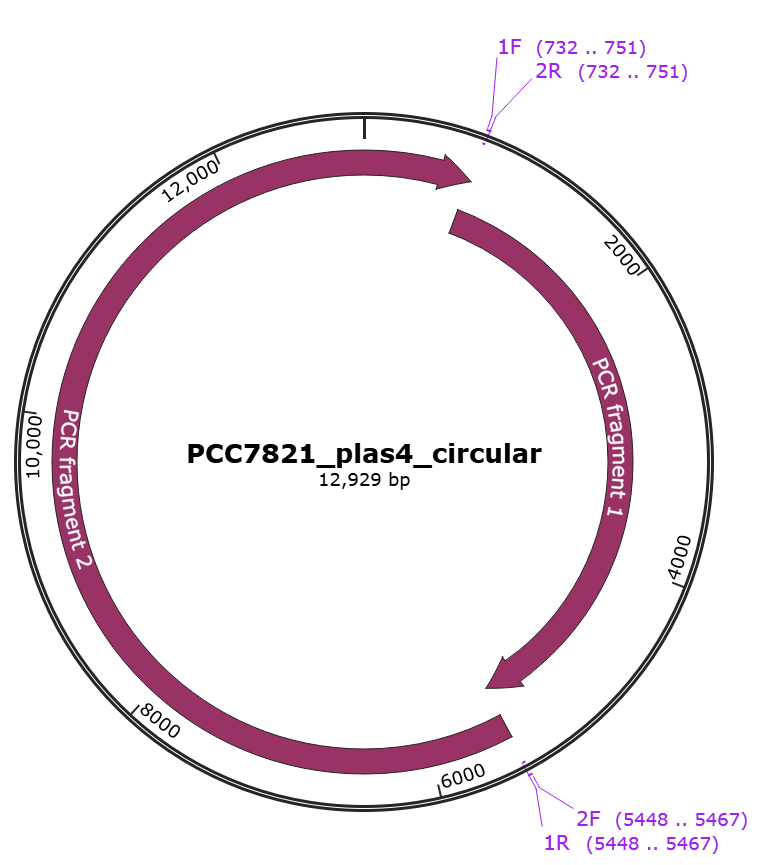 | PCR image (Marker: Generuler 1kb plus)  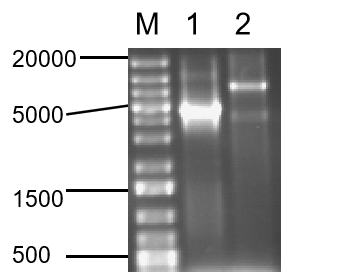 |
| No713_plasmid  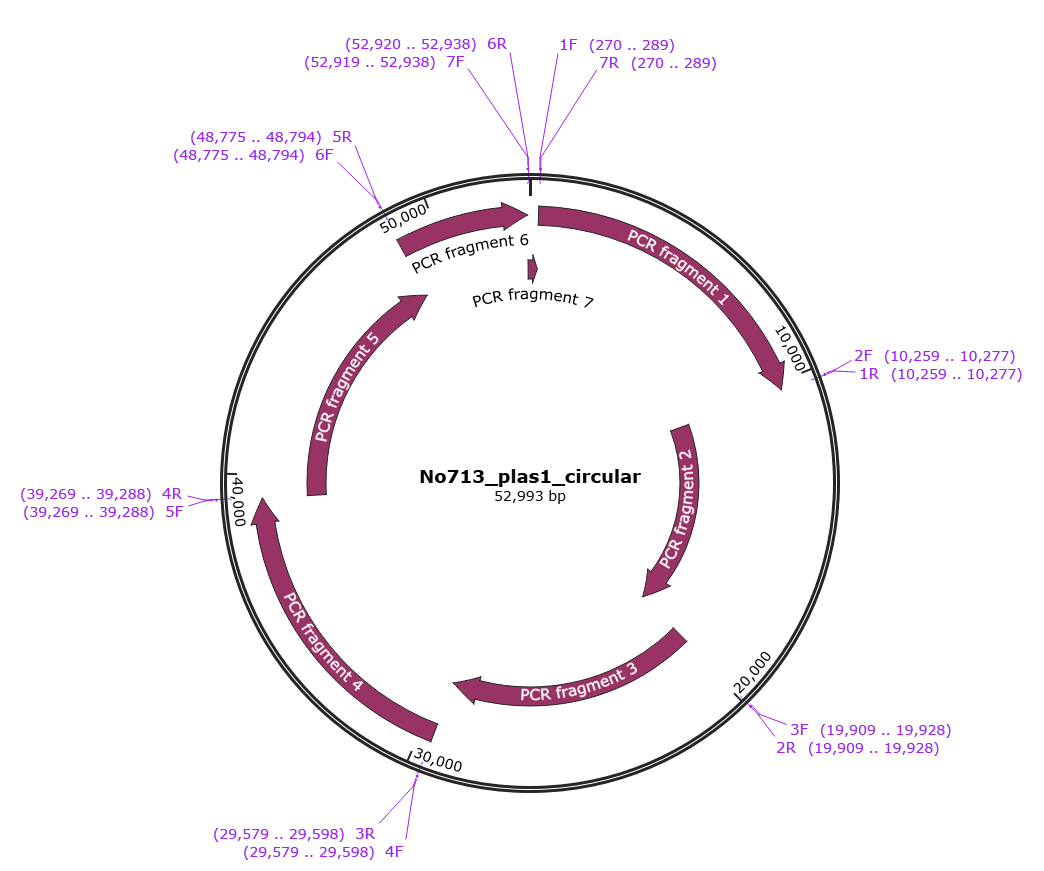 | PCR image (Marker: λ PstI)  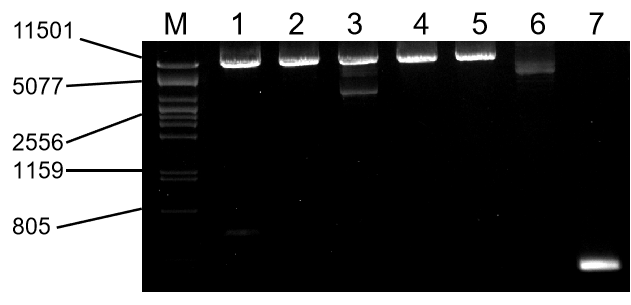 |
| PCC9214_plasmid 1  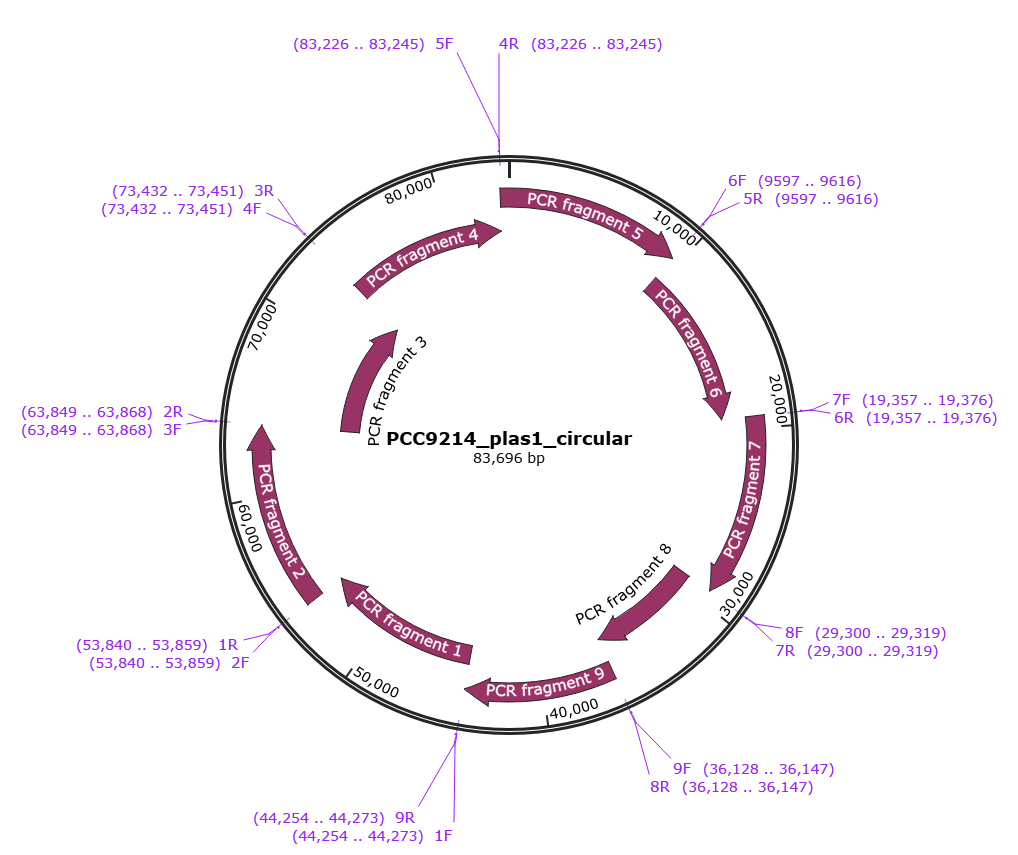 | PCR image (Marker: Generuler 1kb plus)  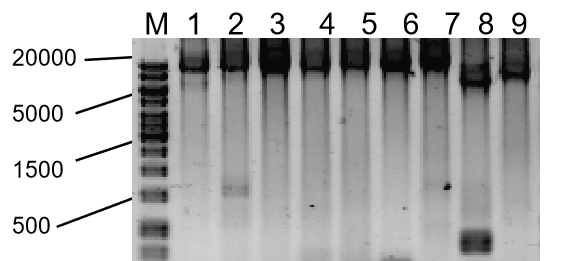 |
| PCC9214_plasmid 2  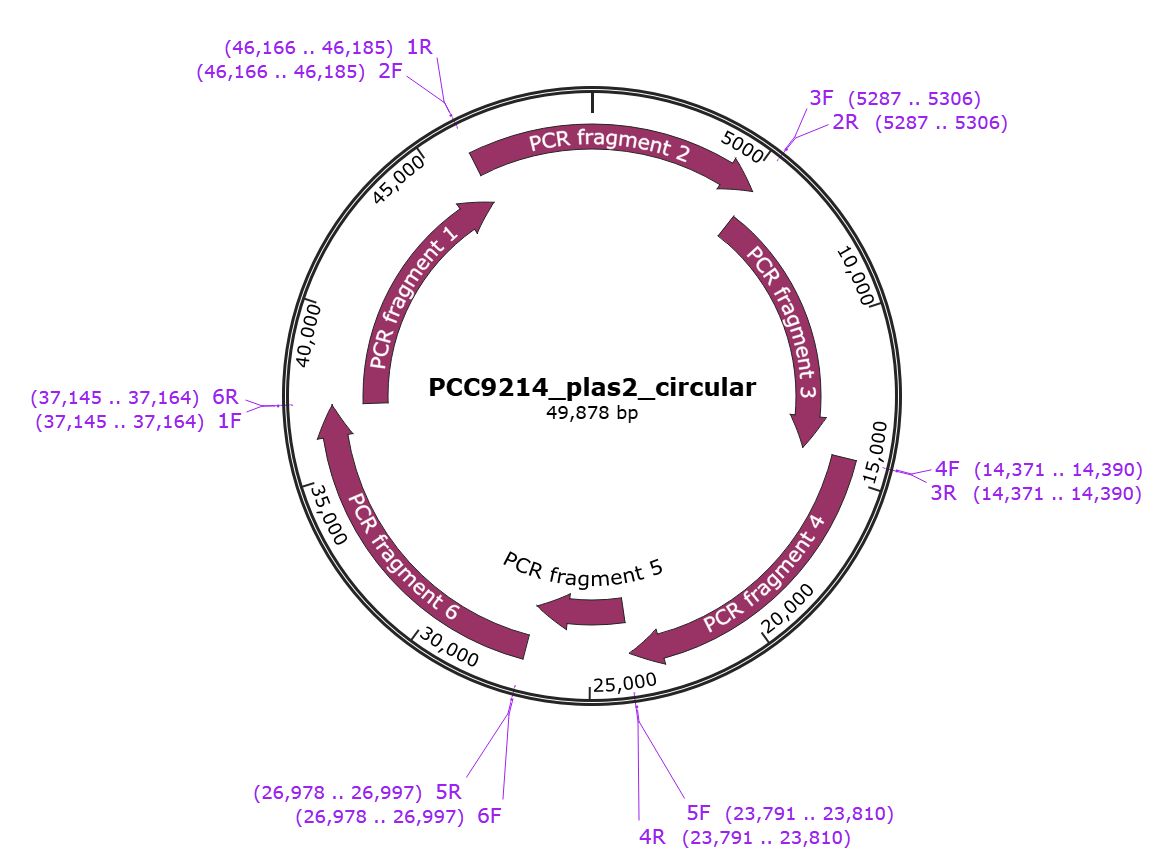 | PCR image (Marker: Generuler 1kb)  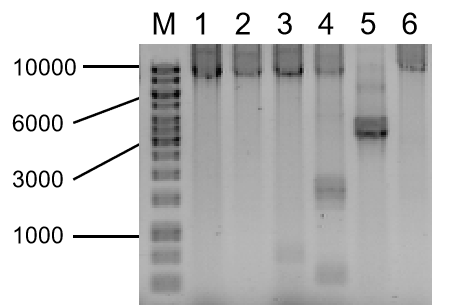 |
