# Supplementary material for: Toxic/Bioactive Peptide Synthesis Genes Rearranged by Insertion Sequence Elements Among the Bloom-Forming Cyanobacteria Planktothrix
Source: Front Microbiol. 2022 Jul 28;13:901762. doi: 10.3389/fmicb.2022.901762 (PMC9366434; doi:10.3389/fmicb.2022.901762)
Supplement: Supplementary file 3 [file Data_Sheet_3.docx]

**Toxic/Bioactive Peptide Synthesis Genes Rearranged by Insertion Sequence Elements Among the Bloom-forming Cyanobacteria *Planktothrix***

Front. Microbiol.

Sec. Evolutionary and Genomic Microbiology

doi: 10.3389/fmicb.2022.901762

**Elisabeth Entfellner^1†^, Ruibao Li^1,2,3†^, Yiming Jiang^2^, Jinlong Ru^2^, Jochen Blom^4^, Li Deng^2^, Rainer Kurmayer^1*^**

^1^ Research Department for Limnology, University of Innsbruck, Mondsee, Austria

^2^ Institute of Virology, Helmholtz Zentrum München, München, Germany

^3^ Department of Ecology and Institute of Hydrobiology, Jinan University, Guangzhou, China

^4^ Bioinformatics and Systems Biology, Justus-Liebig-University, Giessen, Germany

^†^ These authors have contributed equally to this work and share first authorship

*** Correspondence:**Rainer Kurmayer
[rainer.kurmayer@uibk.ac.at](mailto:rainer.kurmayer@uibk.ac.at)

**Additional File 3:**

**Figure S1.** Core (white) and pan (grey) genome development plots calculated via EDGAR 3.0. A) All 13 *Planktothrix* spp. strains; B) *P. agardhii* and *P. rubescens* strains only. (C, D) Core and pan genome developmental plots for (C) all 13 strains or (D) Lineage 1 and 2 arranged according to their phylogenomic relationship in Fig. 1.

**Figure S2** (A). Average amino acid identity (AAI) as calculated from core genes among 13 *Plankothrix* spp. strains. (B). Mean average nucleotide identity (ANI) calculated from 13 *Planktothrix* genomes (using EDGAR 3.0). *Kamptonema* (*Oscillatoria*) PCC6506 was used as outgroup.

**Figure S3**. Synteny plots comparing chromosomal gene arrangements between strains.

**Figure S4** (A). Venn diagrams for chromosomes of 13 strains phylogenetically assigned to one of the three phylogenetic Lineages 1 (n=7), 2 (n=4), 3 (n=2). (B). Percentage of gene functions (COGs) for disjunct core and pan genes compared between phylogenetic Lineages 1 (221 core vs. 1140 pan genes), 2 (418 vs. 1273), 3 (1471 vs. 2001).

**Figure S5**. Schematic view of seven SM synthesis gene clusters recorded from eleven *P. agardhii/P. rubescens* strains (Lineages 1 and 2): A) aeruginosin (*aerA-N*) biosynthesis gene cluster; B) anabaenopeptin (*apnA-E*) biosynthesis gene cluster; C) cyanopeptolin (*ociA-D*) biosynthesis gene cluster; D) microcystin (*mcyA-T*) biosynthesis gene cluster; E) microginin (*micA-E*) biosynthesis gene cluster; F) microviridin (*mvdA-H*) biosynthesis gene cluster; G) prenylagaramide/planktocyclin (*pagA-F*) biosynthesis gene cluster.

**Figure S6**. Maximum Likelihood phylogenetic tree calculated from *pagF* gene sequences (903-927 bp) from eight *P. agardhii/P. rubescens* strains encoding the accessory enzyme prenyltransferase putatively catalyzing post-translational prenylation of the peptide product.

**Figure S7**. Circular plots of the chromosomes showing the location of IS elements and seven SM synthesis gene clusters recorded from *P. agardhii/P. rubescens* (Lineage 1: No2A, No976, PCC7811, PCC7805; Lineage 2: No108, PCC7821).


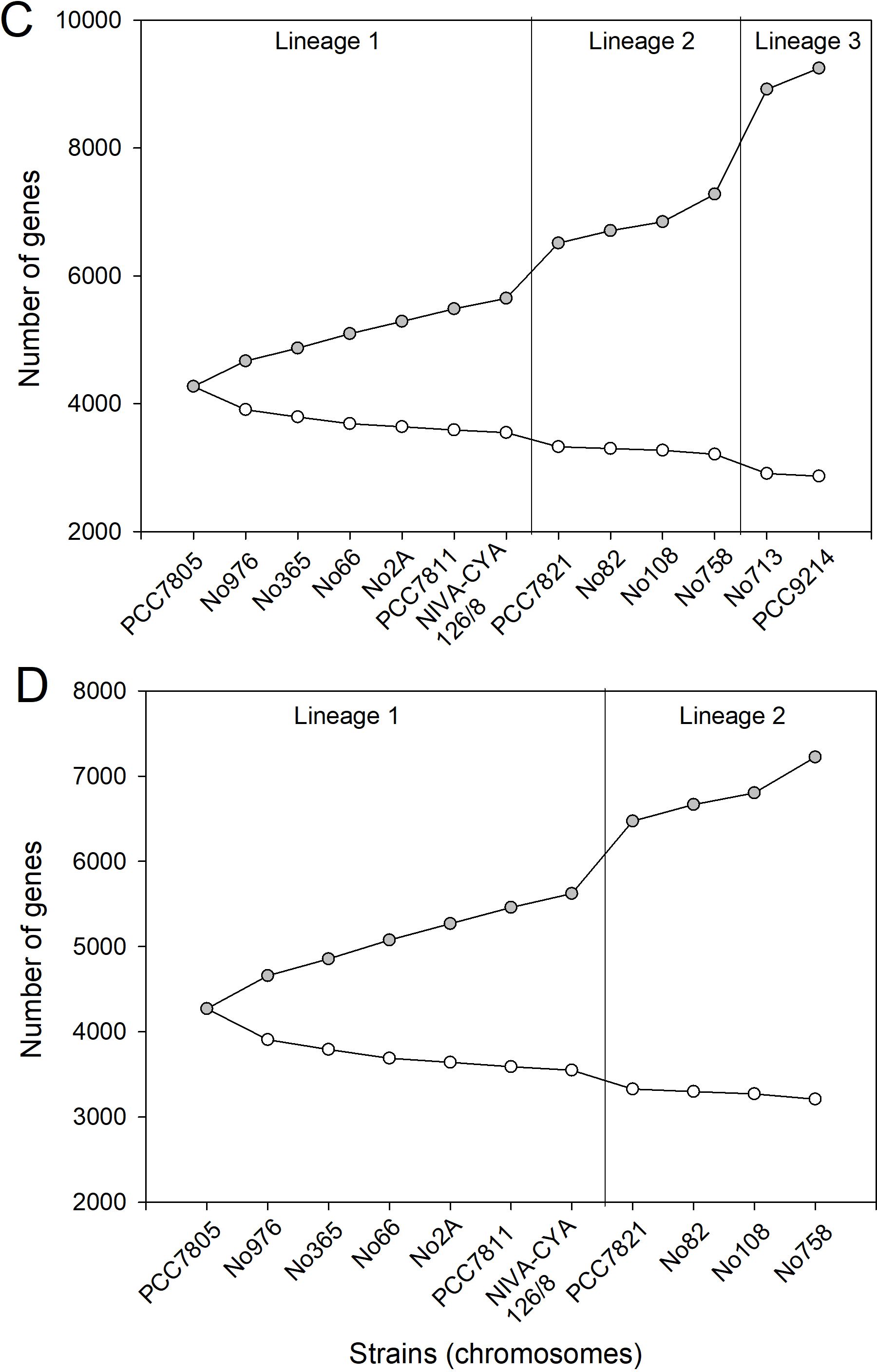


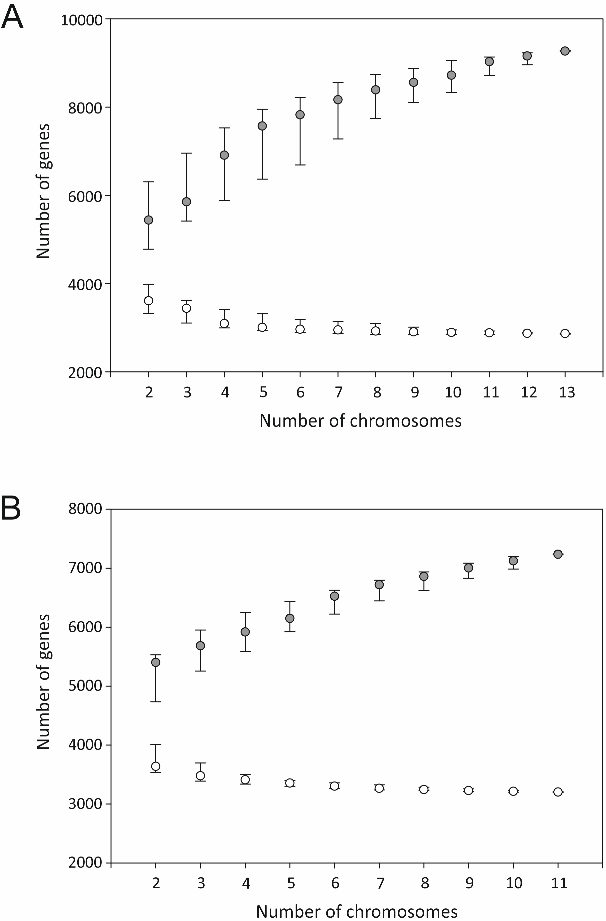


**Figure S1.** Core (white) and pan (grey) genome development plots calculated via EDGAR 3.0. A) All 13 *Planktothrix* spp. strains; B) *P. agardhii* and *P. rubescens* strains only. (C, D) Core and pan genome developmental plots for (C) all 13 strains or (D) Lineage 1 and 2 arranged according to their phylogenomic relationship in Fig. 1.


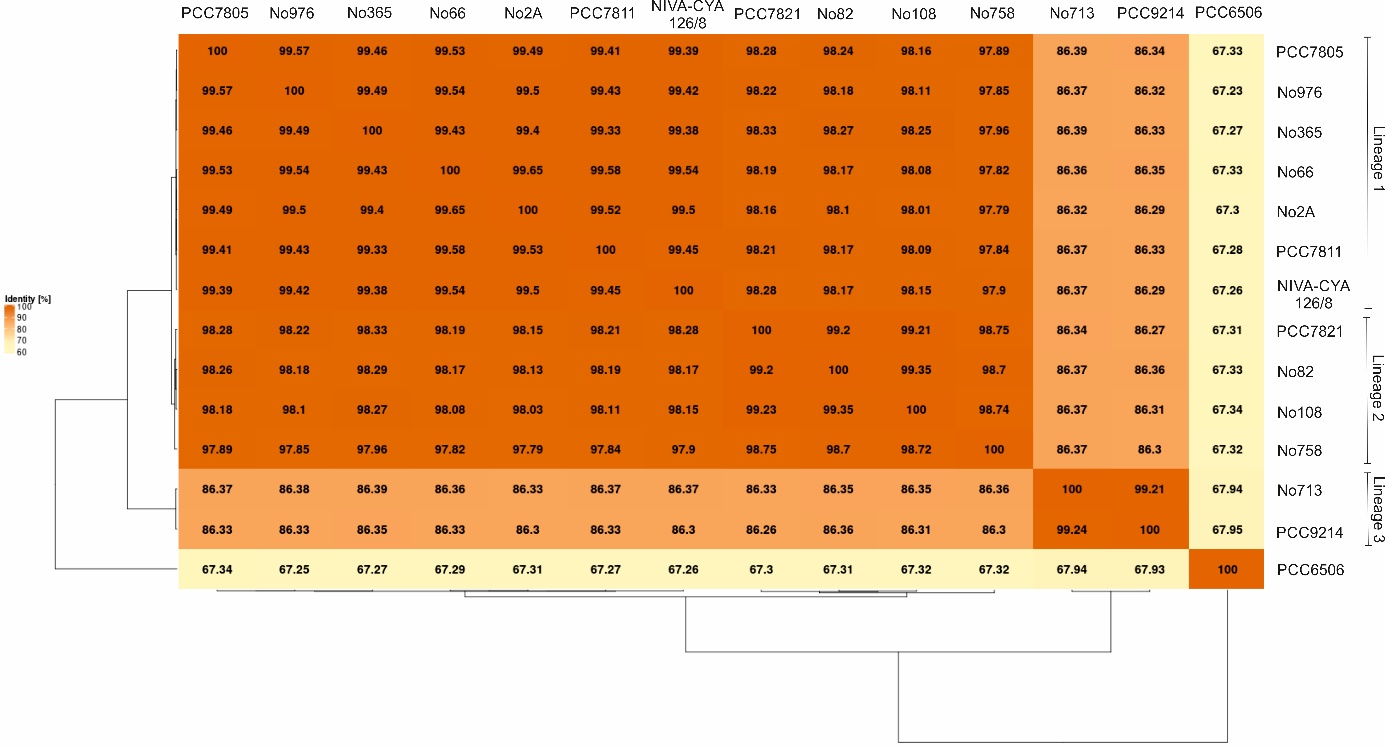


**Figure S2A.** Average amino acid identity (AAI) as calculated from core genes among 13 *Plankothrix* spp. strains. Assignment of strains to phylogenetic lineages as described by Entfellner et al. (2017). *Kamptonema* (*Oscillatoria*) strain PCC6506 was used as an outgroup.


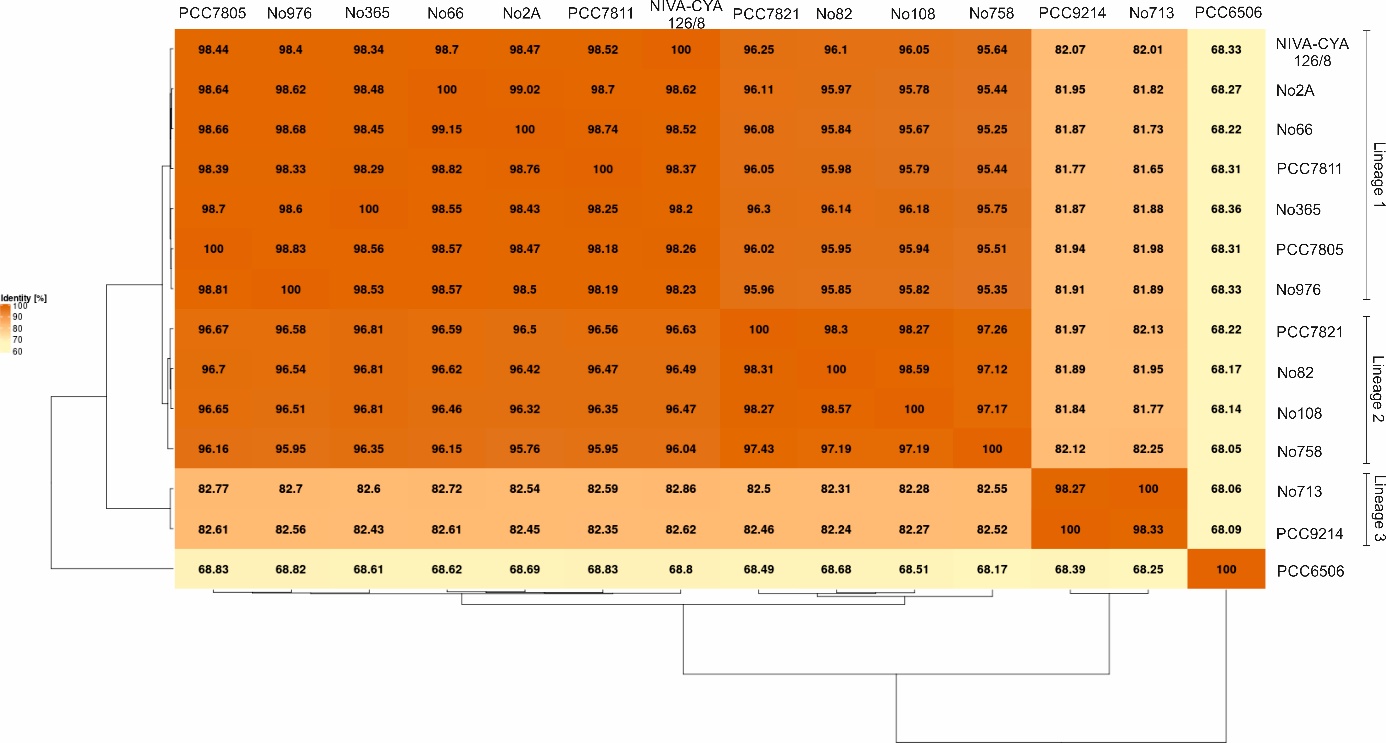


**Figure S2B.** Mean average nucleotide identity (ANI) calculated from 13 *Planktothrix* genomes (using EDGAR 3.0). *Kamptonema* (*Oscillatoria*) PCC6506 was used as outgroup.


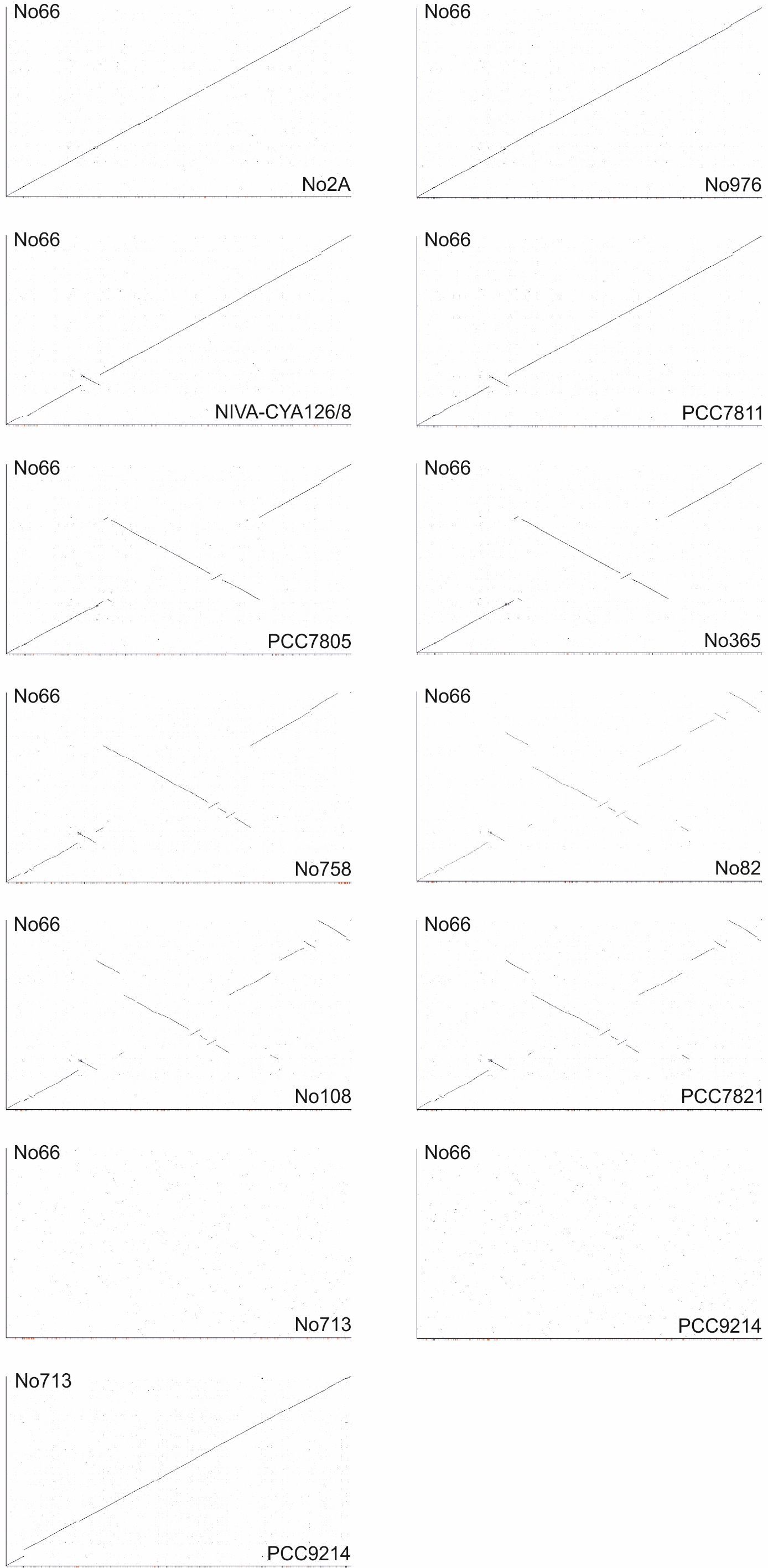

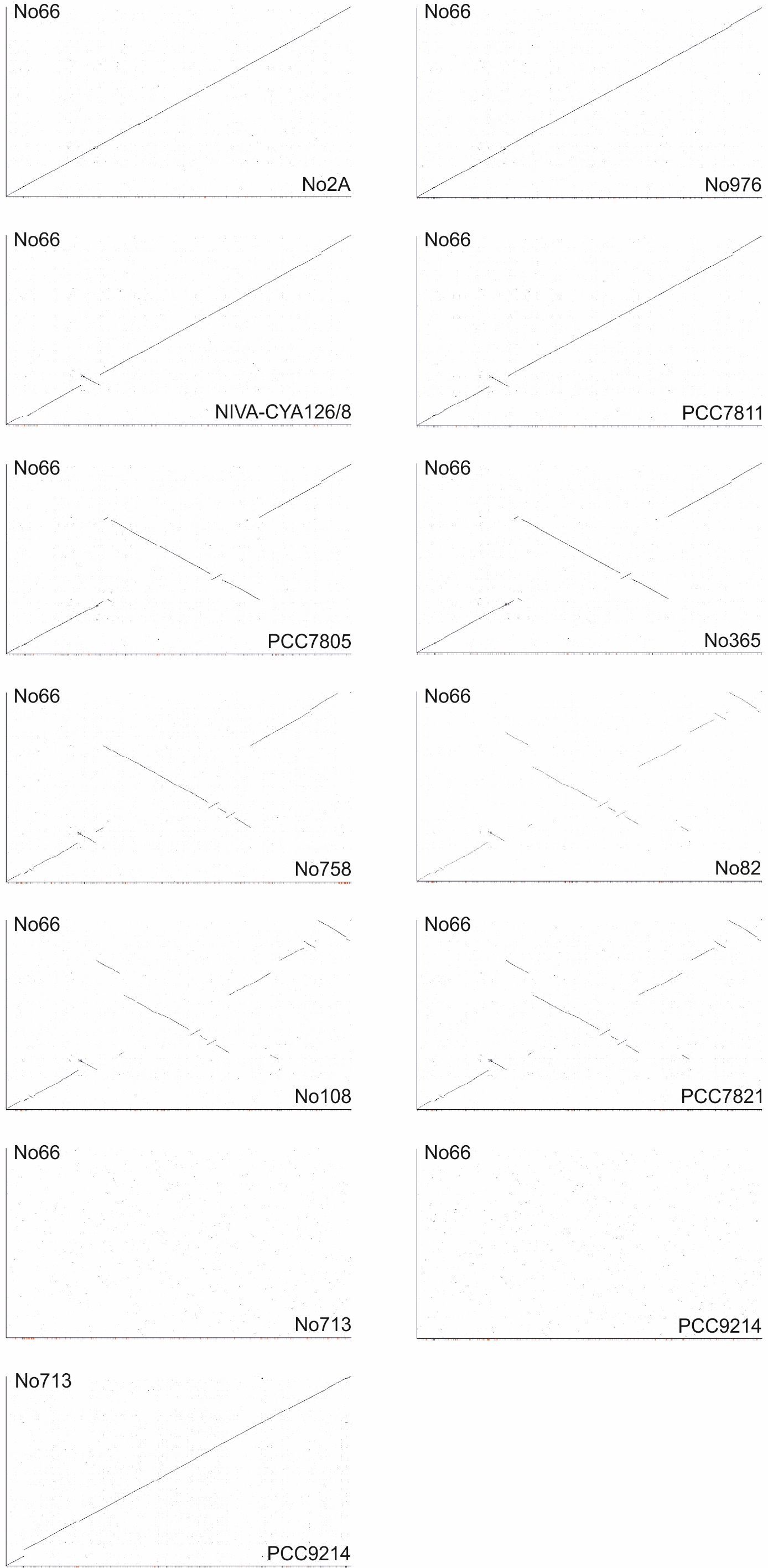


**Figure S3**. Synteny plots comparing chromosomal gene arrangements between strains.


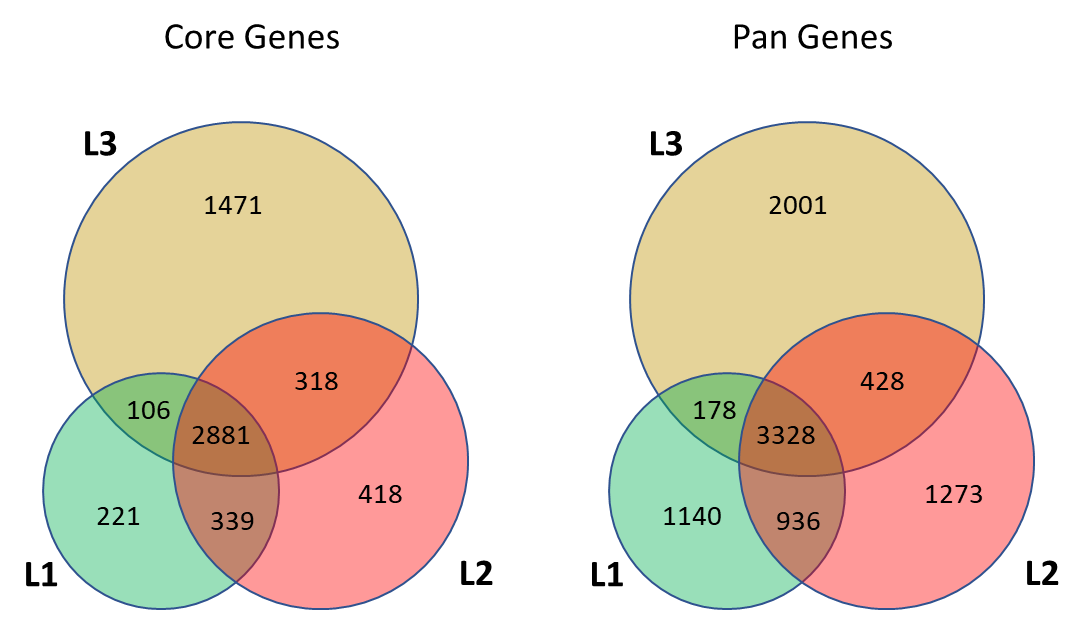


**Figure S4A.** Venn diagrams for chromosomes of 13 strains phylogenetically assigned to one of the three phylogenetic Lineages 1 (n=7), 2 (n=4), 3 (n=2). The diameter of the circle is related to the observed range in chromosome size: L1, Lineage 1 (4.7-4.8 kbp), L2, Lineage 2 (5.4-5.6 kbp), L3, Lineage 3 (6.2-6.6 kbp).

**Figure S4B**. Percentage of gene functions (COGs) for disjunct core and pan genes compared between phylogenetic Lineages 1 (221 core vs. 1140 pan genes), 2 (418 vs. 1273), 3 (1471 vs. 2001). Abbreviations COG categories: J, Translation; K, Transcription; L, Replication and repair (excluding transposases); D, Cell cycle control; V, Defense mechanisms; T, Signal transduction mechanisms; M, Cell wall/membrane/envelope biogenesis; N, Cell motility; U, Intracellular trafficking and secretion; O, Posttranslational modification, protein turnover, chaperone functions; C, Energy production + conversion; G, Carbohydrate transport + metabolism; E, Amino acid transport + metabolism; F, Nucleotide transport + metabolism; H, Coenzyme transport + metabolism; I, Lipid transport + metabolism; P, Inorganic ion transport + metabolism; Q, Secondary metabolite synthesis; R, General function prediction only; S, Function unknown; X, Mobilome: prophages, transposons (incl transposases). COG general categories: J-L, information storage and processing, D-O, cellular processing and storage, C-Q, metabolism and R-X, poorly characterized.


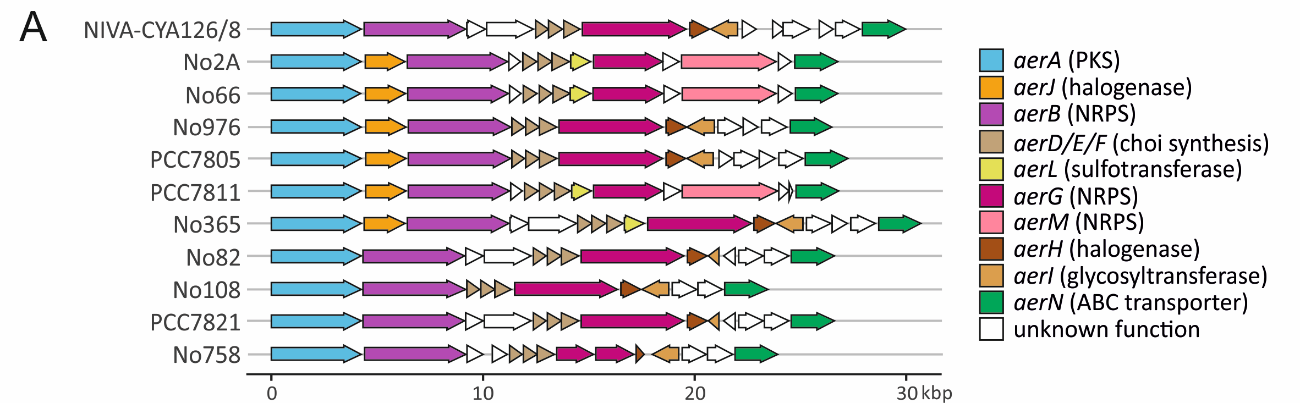


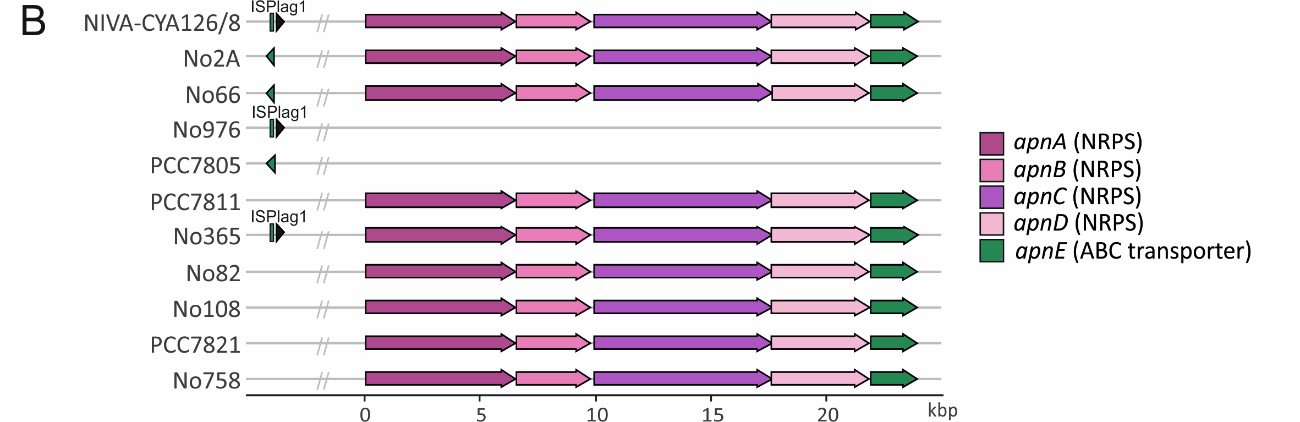


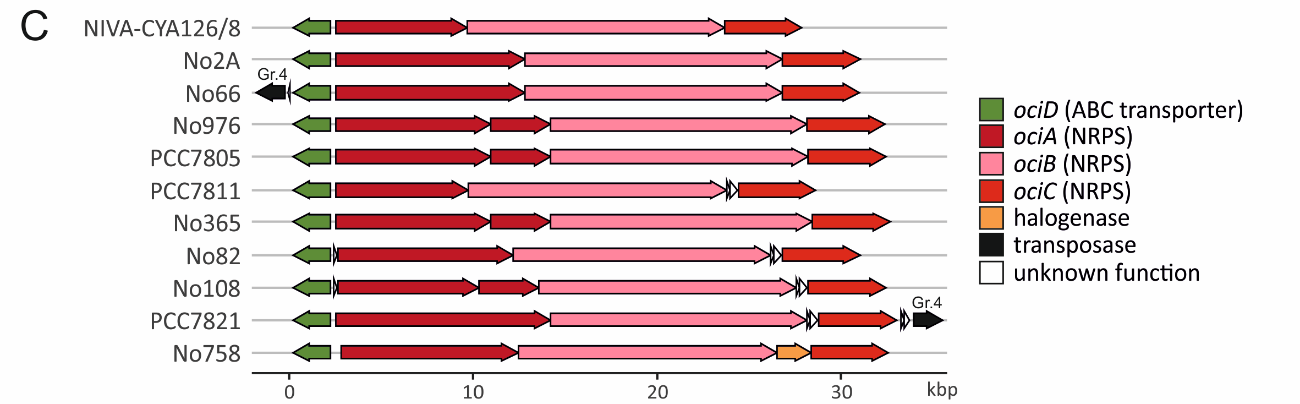


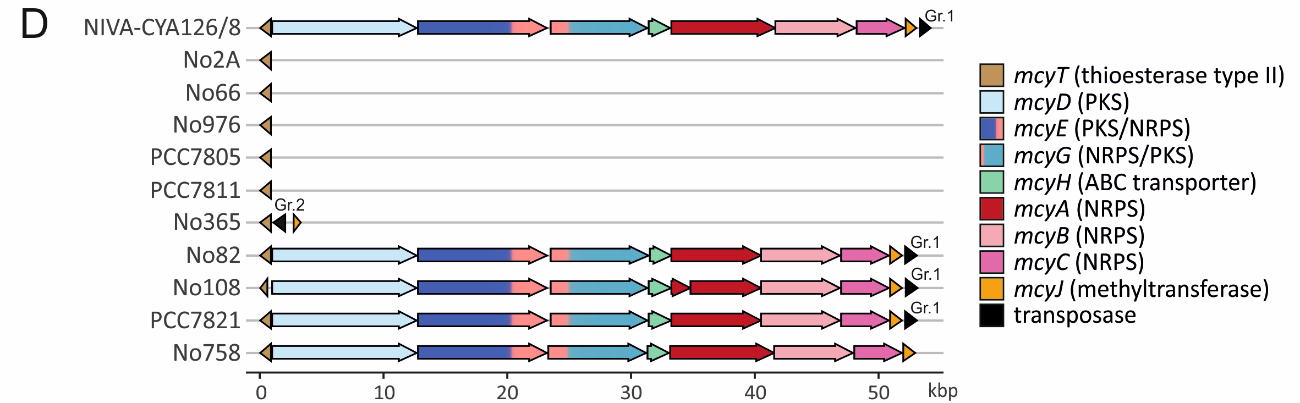


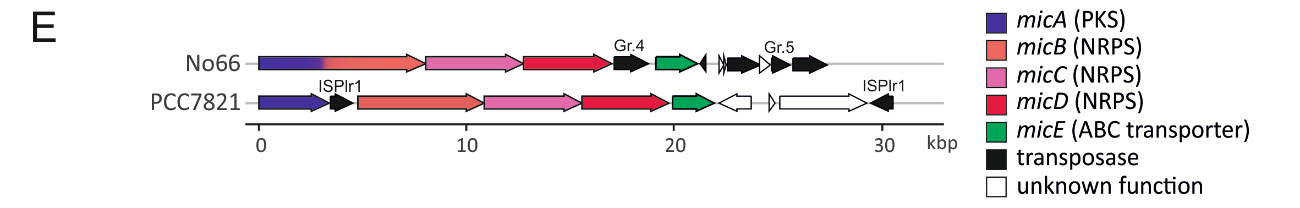


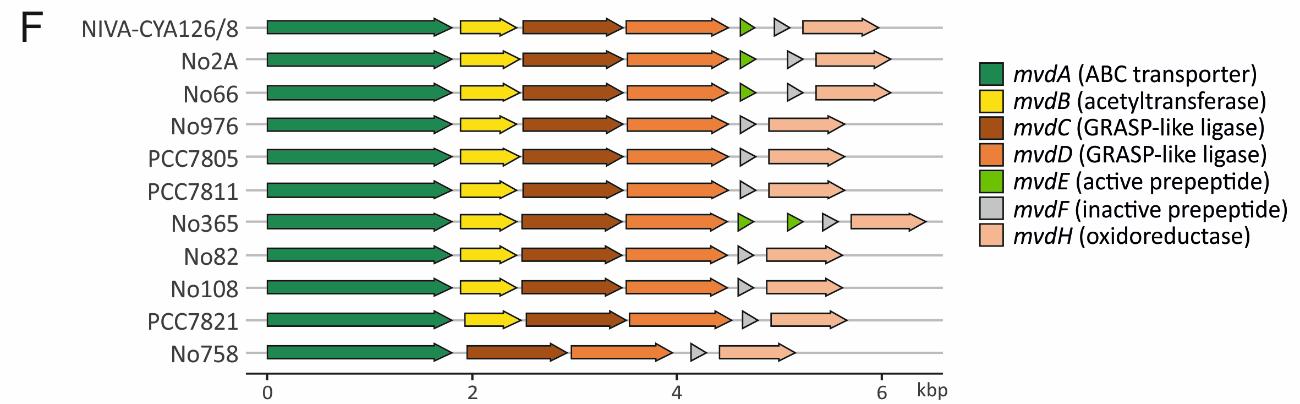


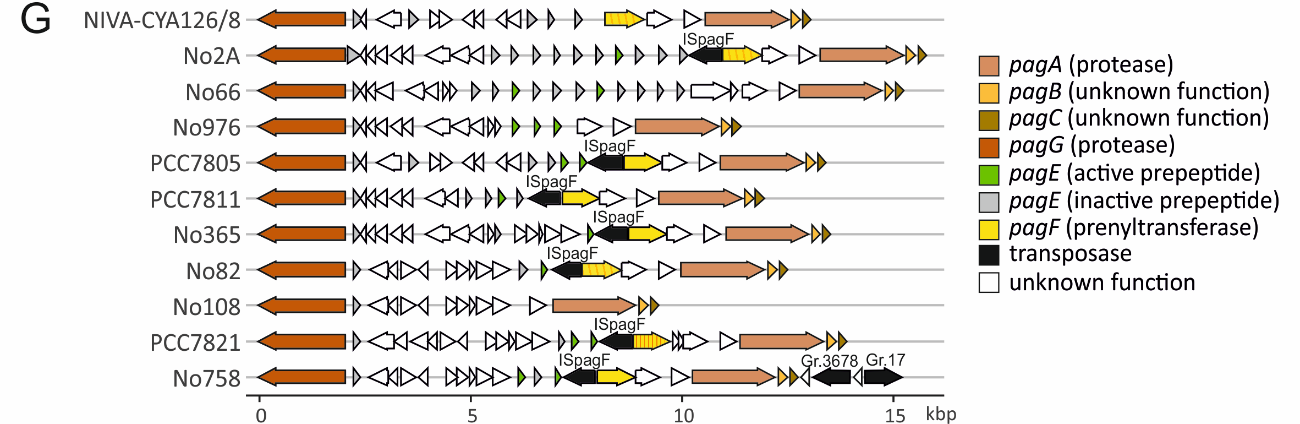


**Figure S5**. Schematic view of seven SM synthesis gene clusters recorded from eleven *P. agardhii/P. rubescens* strains (Lineages 1 and 2): A) aeruginosin (*aerA-N*) biosynthesis gene cluster; B) anabaenopeptin (*apnA-E*) biosynthesis gene cluster; C) cyanopeptolin (*ociA-D*) biosynthesis gene cluster; D) microcystin (*mcyA-T*) biosynthesis gene cluster; E) microginin (*micA-E*) biosynthesis gene cluster; F) microviridin (*mvdA-H*) biosynthesis gene cluster; G) prenylagaramide/planktocyclin (*pagA-F*) biosynthesis gene cluster.

**Figure S6**. Maximum Likelihood phylogenetic tree calculated from *pagF* gene sequences (903-927 bp) from eight *P. agardhii/P. rubescens* strains encoding the accessory enzyme prenyltransferase putatively catalyzing post-translational prenylation of the peptide product.


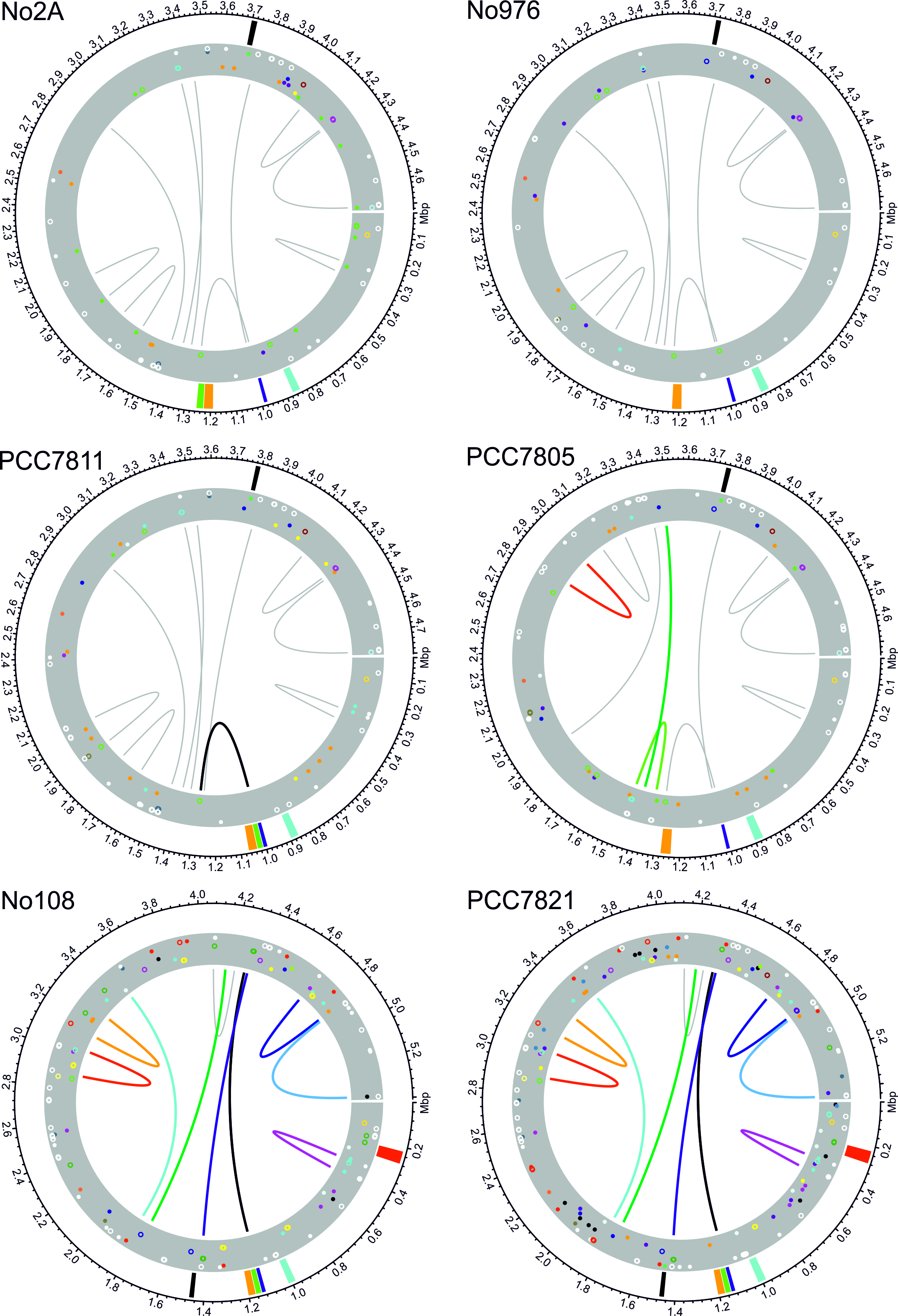


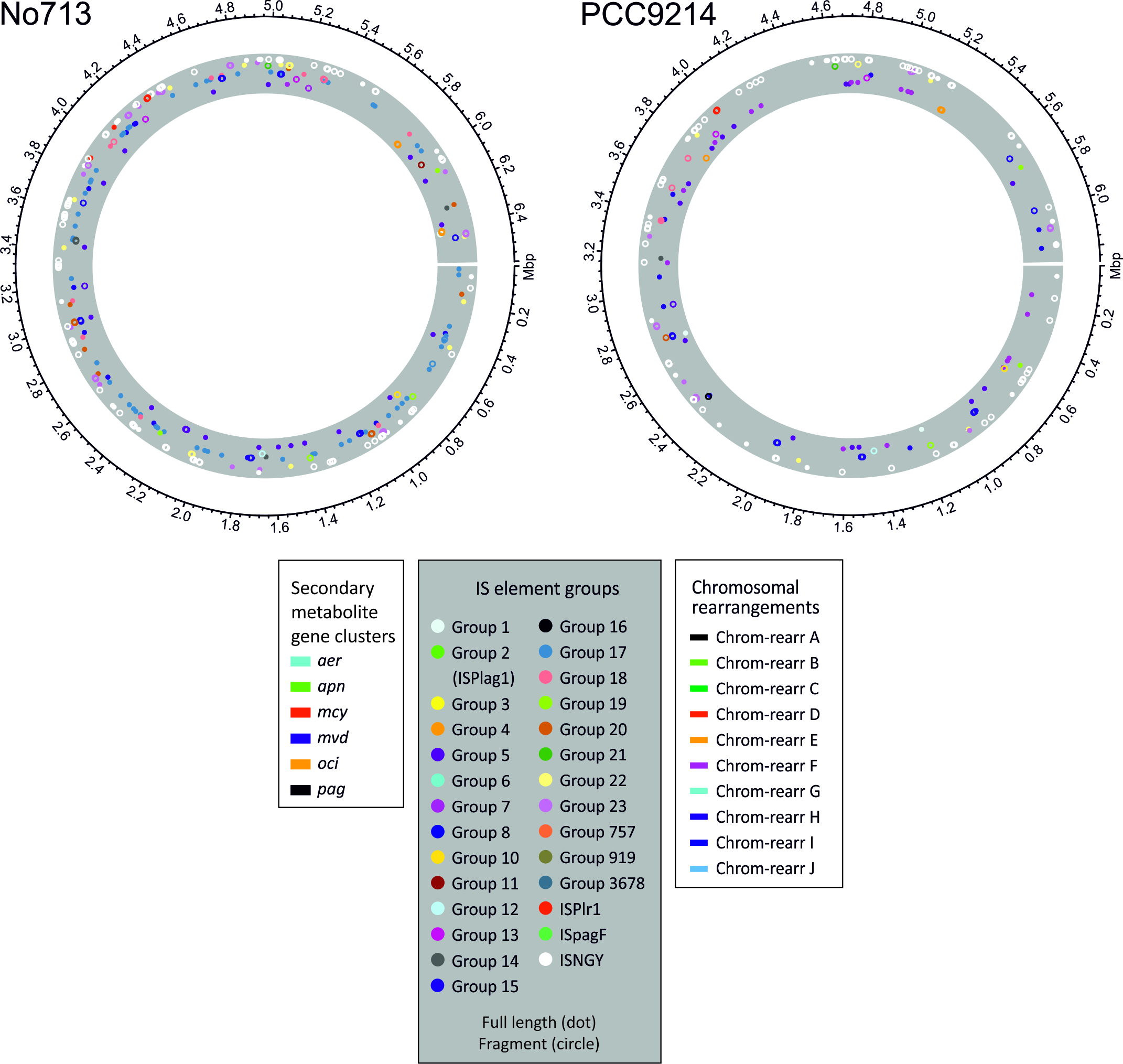


**Figure S7**. Circular plots of the chromosomes showing the location of IS elements and seven SM synthesis gene clusters recorded from *P. agardhii/P. rubescens* (Lineage 1: No2A, No976, PCC7811, PCC7805; Lineage 2: No108, PCC7821). None of the SM gene clusters shown in Fig. S5 were detected in *P. pseudagardhii* (No713) and *P. tepida* (PCC9214). Putative breaking regions of chromosomal rearrangements are indicated by drawn lines.
